# Ceramide Kinase (CERK) Emerges as a Common Therapeutic Target for Triple Positive and Triple Negative Breast Cancer Cells

**DOI:** 10.3390/cancers14184496

**Published:** 2022-09-16

**Authors:** Kajal Rajput, Mohammad Nafees Ansari, Somesh K. Jha, Trishna Pani, Nihal Medatwal, Somdeb Chattopadhyay, Avinash Bajaj, Ujjaini Dasgupta

**Affiliations:** 1 Amity Institute of Integrative Sciences and Health, Amity University Haryana, Panchgaon, Manesar, Gurgaon 122413, Haryana, India; 2 Regional Centre for Biotechnology, NCR Biotech Science Cluster, 3rd Milestone Faridabad-Gurgaon Expressway, Faridabad 121001, Haryana, India

**Keywords:** breast cancer, sphingolipids, triple-negative breast cancer, triple-positive breast cancer, ceramide-1-phosphate, siRNA delivery, localized therapy

## Abstract

**Simple Summary:**

Existing chemotherapy treatments for breast cancer patients are high on toxicity. There are very limited options available for triple-positive breast cancer (TPBC) patients, and there have not been any major breakthrough for targeted therapy for triple-negative breast cancer (TNBC) patients. Therefore, there is a need to identify common therapeutic targets for breast cancer patients. In this manuscript, we compared the sphingolipid profiles of cancer cell lines representing TPBC and TNBC, and correlated these profiles with the proliferation and migration properties the of cell types. We then associated the sphingolipid profiles for each subtype specific cell line with transcriptional and translational expression of corresponding metabolizing enzymes. Our results suggested that ceramide kinase (CERK) that catalyzes the synthesis of ceramide-1-phosphates from ceramides is dysregulated in both cell types. We also showed that the targeting of CERK at transcriptional level by siRNA therapeutics or inhibiting the CERK activity by hydrogel-mediated delivery of chemical inhibitors can be an effective strategy to slow down the tumor progression. Therefore, CERK emerges as a potential therapeutic target that can be explored further for cancer therapy.

**Abstract:**

Sphingolipids are key signaling biomolecules that play a distinct role in cell proliferation, migration, invasion, drug resistance, metastasis, and apoptosis. Triple-negative (ER−PR−HER2−) and triple-positive (ER+PR+HER2+) breast cancer (called TNBC and TPBC, respectively) subtypes reveal distinct phenotypic characteristics and responses to therapy. Here, we present the sphingolipid profiles of BT-474 and MDA-MB-231 breast cancer cell lines representing the TPBC and TNBC subtypes. We correlated the level of different classes of sphingolipids and the expression of their corresponding metabolizing enzymes with the cell proliferation and cell migration properties of BT-474 and MDA-MB-231 cells. Our results showed that each cell type exhibits a unique sphingolipid profile, and common enzymes such as ceramide kinase (CERK, responsible for the synthesis of ceramide-1-phosphates) are deregulated in these cell types. We showed that siRNA/small molecule-mediated inhibition of CERK can alleviate cell proliferation in BT-474 and MDA-MB-231 cells, and cell migration in MDA-MB-231 cells. We further demonstrated that nanoparticle-mediated delivery of CERK siRNA and hydrogel-mediated sustained delivery of CERK inhibitor to the tumor site can inhibit tumor progression in BT-474 and MDA-MB-231 tumor models. In summary, distinct sphingolipid profiles of TPBC and TNBC representing cell lines provide potential therapeutic targets such as CERK, and nanoparticle/hydrogel mediated pharmacological manipulations of such targets can be explored for future cancer therapeutics.

## 1. Introduction

Lipid reprogramming has emerged as a key hallmark for cancer pathogenesis [1,2]. Sphingolipids with prominent structural and signaling roles show altered metabolism during breast cancer progression [3,4]. Diverse regulatory mechanisms by key sphingolipids affect inflammation, angiogenesis, cell proliferation, apoptosis, and drug resistance [5,6]. Multiple factors including the movement of lipid flux, receptor interactions, target gene expression and protein modulations that balance the availability and context-specific function of different bioactive sphingolipids influence the multicomponent “sphingodynamic model” [7,8]. Therefore, profiling sphingolipid species and correlating this with expression of sphingolipid-metabolizing enzymes in different breast cancer subtypes will help in the identification of common targets that can be explored for future therapeutics.

**Figure 1:**
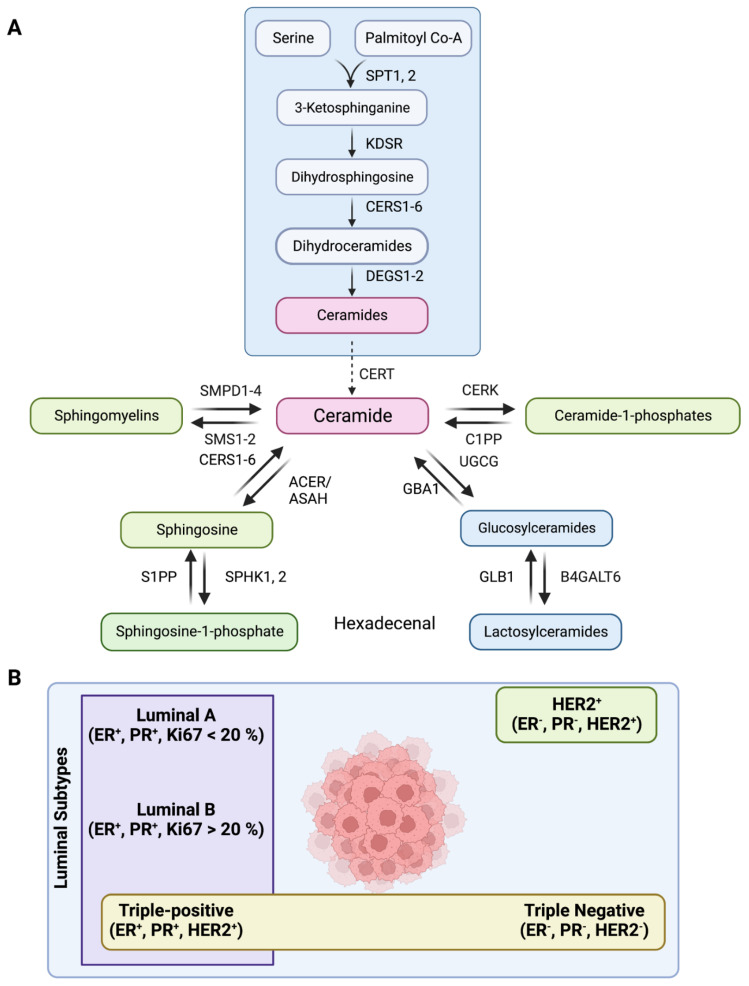
(**A**) Schematic showing the sphingolipid metabolic pathway where serine palmitoyl transferase (SPT1–2), 3–Ketosphinganine reductase (KDSR), Dihydroceramide desaturase (DEGS1–2), Ceramide synthases (CERS1–6), Ceramide transferase (CERT), Ceramide 1-phosphate phosphatase (C1PP), *N*–Acylsphingosine amidohydrolase (ASAH1, ASAH2), Alkaline ceramidase (ACER1, ACER3), Sphingosine–1–phosphate kinase (SPHK1, 2), Sphingosine–1–phosphate phosphatase (S1PP), Sphingosine–1–phosphate lyase (S1PL), Ceramide Kinase (CERK), Sphingomyelin Phosphodiesterase (SMPD1–4), Sphingomyelin synthase (SMS1–2), UDP-Glucose ceramide glucosyltransferase (UGCG), *β*–glucocerebrosidase (GBA1), *β*–galactosidase (GLB1), β–1,4–Galactosyltransferase 6 (B4GALT6) are the enzymes of the pathway. (**B**) Schematic showing major subtypes of breast cancer, such as luminal A, luminal B, luminal B with HER2 overexpression (also called triple-positive, TPBC), triple-negative (TNBC)/Basal-like, and HER2^+^.

Ceramide, the central player in the sphingolipid pathway, induces apoptosis and cell senescence in response to chemotherapy and radiation therapy (Figure 1A) [9,10]. De novo or salvage pathways can generate ceramide-mediated apoptosis, and the availability of cytotoxic ceramides can be restricted by sphingomyelin synthases (SMS1, 2), ceramide kinase (CERK), and ceramidases (Figure 1A) [11,12]. In contrast, elevated levels of sphingosine kinase 1 (SPHK1) and sphingosine-1-phosphate (S1P) are witnessed in various cancer cell lines and tumor tissues where S1P is responsible for enhanced cell proliferation, angiogenesis, and inflammation [13]. Ceramide and S1P act as components of “sphingolipid rheostat or more complex sphingodynamic model” that regulates cell fate, survival, and tissue homeostasis [14]. Interestingly, ceramide-1-phosphate (C1P), the phosphorylated metabolite of ceramide, is also antiapoptotic, and is a regulator of cancer cell proliferation and migration [15,16,17]. Glucosylceramides synthesized from ceramides by UDP-Glucose ceramide glucosyltransferase (UGCG) promote cell proliferation and drug resistance [18], and increased levels of sphingomyelins synthesized from ceramides by SMS 1, 2 are important components of the lipid-raft-mediated signaling processes (Figure 1A) [19]. Therefore, the structural and functional diversity of sphingolipids are responsible for their interactions with specific target proteins that allow them to perform diverse roles during tumor progression [20].

There are five major subtypes of breast cancer based on the expression of estrogen receptor (ER), progesterone receptor (PR), human epidermal growth factor receptor 2 (HER2), and Ki67 (a proliferation marker). These subtypes include luminal A (ER+PR+HER2−, Ki67+ < 20%), luminal B (ER+PR+HER2−, Ki67+ ≥ 20%), luminal B with HER2 overexpression (triple-positive/TPBC) (ER+PR+HER2+), triple-negative (TNBC/Basal-like) (ER−PR−HER2−), and HER2+ (ER−PR−HER2+) (Figure 1B) [21]. Around 70% of breast tumors are of luminal subtype (ER/PR+) [22], where luminal B tumors with higher Ki67 expression show higher proliferation rate, advanced histological grade and worse prognosis than luminal A patients [23]. A smaller subset of luminal B subtype also have high expression of HER2 (around 30% of all HER2^+^ tumors), and are categorized as triple-positive (TPBC) phenotypes [24]. Therefore, simultaneous inhibition of HER2 and hormone receptor pathways is more effective than endocrine therapy alone in TPBC subtype tumors [25]. In contrast, triple-negative (TNBC)/basal-like tumors exhibit very high proliferative and mitotic index, more severe histological and nuclear grade, exceptionally high rate of migration and metastasis, and poor prognosis [26]. Chemotherapy is the preferred line of treatment for TNBC patients as endocrine therapy or other molecular targeted therapies have poor outcome [27,28].

Each breast cancer subtype has a unique molecular portrait that influences its response to therapy, prognosis, and outcome. Earlier studies have reported the sphingolipid profile in luminal (ER+PR+) and TNBC (ER−PR−HER2−) subtypes [29,30]. However, none of the studies have compared the sphingolipid profiles of TPBC and TNBC subtypes and their correlation with the phenotypes manifested by these cells. Here, we present the unique sphingolipid profile of TPBC (ER+PR+HER2+) and TNBC subtype representing cancer cells followed by correlation of their phenotypic (proliferation and migration) characters with sphingolipid levels and expression of corresponding metabolizing genes/enzymes. Based on these molecular associations, we identified and validated ceramide kinase (CERK, responsible for synthesis of ceramide-1-phosphates from ceramides) as a key therapeutic target for TPBC and TNBC subtypes. We further demonstrated that nanoparticle/hydrogel mediated inhibition of CERK using siRNA or chemical inhibitor can be an effective strategy to mitigate cell proliferation and tumor progression in these subtypes.

## 2. Materials and Methods

Materials. Human breast cancer cell lines BT-474 and MDA-MB-231 were purchased from American Type Culture Collection (ATCC, Manassas, VA, USA). DMEM (Cat#D5648), DPBS (Cat#D5652), Crystal Violet Dye (Cat#C0775) were purchased from Sigma–Aldrich^®^, Saint Louis, MO, USA. Fetal bovine serum (Cat#10270) was purchased from, Waltham, MA, USA. Matrigel (Cat#354234) was purchased from Corning Inc., Bedford, MA, USA. Penicillin-Streptomycin solution (SV30079) was purchased from Cytiva HyClone^TM^, South Logan, OH, USA. Trypsin (Cat#TCL007), MEM Medium (Cat#AL081) and sodium bicarbonate (GRM849) were purchased from HiMedia Laboratories, Mumbai, India.

Lipofectamine 2000 (Cat#11668019) and TURBO^TM^ DNase (Cat#AM2238) was from Invitrogen, Vilnius, Lithuania. iScript^TM^ cDNA synthesis kit (Cat#1708891) and iTaq™ universal SYBR^®^Green Supermix (Cat#1725124) were purchased from Bio-Rad Laboratories, Hercules, CA, USA. RNase Inhibitor (Cat#AM2694), and Pierce^TM^ BCA protein assay kit (Cat#23227) were purchased from Thermo Scientific. Complete™, Rockford, IL, USA. Protease Inhibitor Cocktail (P8340-1ML) was purchased from Sigma–Aldrich^®^, Saint Louis, MO, USA. Sigma. CerK siRNA (Cat#L-004061-00-0005) and scrambled siRNA (Cat# D-001810-10-05) were purchased from Dharmacon Inc., Cambridge, UK. Ceramide Kinase Inhibitor, NVP231 (Cat#3960) was purchased from Tocris Bioscience, Bristol, UK.

Ceramide/Sphingoid Internal Standard Mixture II (Cat#LM6005-1EA) was purchased from Avanti^®^ Polar Lipids, Inc., Alabaster, OK, USA. LASS1 (CERS1) (Cat#H00010715-A01), LASS2 (CERS2) (Cat#H00029956-M01A), LASS4 (CERS4) (Cat#H00079603-M01), LASS6 (CERS6) (Cat#H00253782-M01) were purchased from Abnova, Taipei, Taiwan. LASS5 (CERS5) (Cat#ab73289), CerK (Cat#ab155061), SPHK1 (Cat#ab109522), SPHK2 (Cat#ab264042), A-SMase (SMPD1) (Cat#ab83354), N-SMase1 (SMPD2) (Cat#ab131330), N-SMase2 (SMPD3) (Cat#ab199399), N-SMase3 (SMPD4) (Cat#ab133935), UGCG (Cat#ab124296), SMS1 (Cat#ab135365), SMS2 (Cat#ab237681), GBA1 (Cat#ab88300), GLB1 (Cat#ab96239), B4GALT6 (Cat#ab200639), Goat anti-mouse IgG (L+H) (Cat#ab6789) were purchased from Abcam, Cambridge, UK. ASAH1 (Cat#HPA005468), ASAH2 (Cat#ABS457), *β*-actin (Cat#A5541) were purchased from Sigma–Aldrich^®^, Saint Louis, MO, USA. Goat anti-rabbit IgG-HRP was purchased from (Cat#sc-2004) Santa Cruz Biotechnology, Inc., Dallas, TX, USA. 

Nitrocellulose (Cat#HATF00010), Immobilon Western Chemiluminescent HRP substrate (Cat#WBKLS0500), ethanol (Cat#100983), glacial acetic acid (Cat#193402), chymotrypsin (11418467001), DMSO (Cat#276855), ethidium bromide (Cat#E8751), agarose (Cat#A9539), magnesium chloride (Cat#208337), sodium dodecyl sulfate (Cat#L3771), ammonium persulfate (Cat#A3678), *N*,*N*′-methylene bisacrylamide (Cat#M7279), glycine (Cat#G8898), MTT (Cat#M5655), triethyl ammonium bicarbonate buffer (Cat#T70408), and iodoacetamide (144-48-9) were purchased from Sigma–Aldrich^®^, Saint Louis, MO, USA. Tris (Cat#MB029), NaCl (Cat#GRM853), polyoxyethylenesorbitan monolaurate (Tween^®^ 20) (Cat#P7949), glycerol (Cat#GRM1027), bovine serum albumin fraction-V (Cat#GRM105) were purchased from HiMedia Laboratories, Mumbai, India. Paraformaldehyde (PFA) (Cat 81847) was purchased from Thomas Baker, Mumbai, India. Ammonium hydroxide (Cat#16227) and hydrochloric acid (Cat#29505) was purchased from Thermo Fisher Scientific, Waltham, MA, USA. MOPS free acid (Cat#MB0360) and acrylamide (Cat#AB1032) were purchased from Bio Basics Inc., Markham, ON, Canada. Sodium hydroxide (Cat#13913) and potassium hydroxide (Cat#84749) was purchased from Sisco Research Laboratories Pvt. Ltd., Maharashtra, India. Developer (Cat#4908216), Fixer (Cat#4908232) and XBT X-Ray film (Cat#6568307) were purchased from Carestream, Rochester, NY USA. Transwell Migration Plate (Cat#3464) was purchased from Corning Inc., Bedford, MA, USA. 

Methanol (Cat#34966), chloroform (Cat#25669-1L), 2-propanol (Cat#34965), acetonitrile (Cat#34967), ammonium acetate (Cat#14267-25G), formic acid (Cat#56302-50ML), water (Cat#39253-4L) and ammonium formate (Cat#14266-25G) were purchased from Honeywell International Inc., Charlotte, NC, USA. ACQUITY UPLC BEH Shield RP18 column (Cat#186002854) was purchased from Waters™ Ltd., Milford, MA, USA.

Cell culture. Human breast cancer cell lines BT-474 and MDA-MB-231 were cultured in DMEM high glucose media with 10% Fetal bovine serum, 100 units/mL penicillin, and 100 µg/mL streptomycin. Cells were grown at 37 °C with 5% CO_2_ in humidified incubator. 

siRNA transfections in cell culture. BT-474 and MDA-MB-231 cells were transfected with siRNA (targeting CERK or scrambled) using Lipofectamine 2000. BT-474 and MDA-MB-231 cells (~3.5 × 10^5^ per well) were seeded in six-well plate in DMEM media with 10% FBS and 10% penicillin and streptomycin, and incubated at 37 °C in CO_2_ incubator for 24 h. At 80–85% confluency, siRNA-lipofectamine complexes in 1:3 ratio were incubated for 25 min in MEM media, and cells were transfected with these complexes. After 6 h of transfection, media was removed, and cells were incubated with antibiotic-free media containing 10% FBS for 36 h. CERK silencing was confirmed by Western blotting.

In vitro assays. For proliferation assay, BT-474 and MDA-MB-231 cells or siRNA transfected cells (scrambled or CERK siRNA) (5000 cells/well) were used, and cell proliferation assay was performed following previously described method [31]. 

For transwell migration assay, BT-474 and MDA-MB-231 cells or siRNA transfected cells (scrambled or CERK siRNA) transfected cells (60,000 cells) were seeded in transwell inserts (4 µm pore size). We then placed the inserts in 24-well cell culture plates containing DMEM with 10% FBS and incubated for 24 h at 37 °C. We fixed the migrated cells in 4% paraformaldehyde (PFA) (~5 min), and permeabilized with methanol (20 min). Cells were then stained with 2% crystal violet dye (15 min) followed by PBS washing to remove the extra stain on the cell surface. Cells were counted manually and imaged using a Nikon microscope. 

Pellet collection for RNA and protein isolation. For RNA isolation, cells were grown in 100 mm cell culture plates for 90% confluency. For pellet collection, media was aspirated from the plates and washed two times with DPBS. Trizol (1 mL) was added to the plate, and plates were incubated for 5 min. After incubation, cells were scraped and transferred into 1.5 mL centrifuge tubes, and were used immediately or stored at −80 °C. For protein isolation, cells were similarly washed, scraped out in DPBS, centrifuged at 5000 rpm for 5 min, and used immediately or stored at −80 °C.

Quantitative Real-Time PCR. Total RNA extraction and qRT-PCR studies were performed using previously described method [31]. All primer sequences used for RT PCR are listed in Table S1. 

Western Blotting. Protein expression analysis was performed by Western blotting as per previously described method [31]. Protein separation was performed on 10–12% SDS-PAGE using 15–30 μg of protein. After separation, protein was transferred to nitrocellulose membrane. Immunostaining was performed by overnight incubation of proteins with corresponding primary antibody at 4 °C in 5% BSA/Skimmed milk in TBST. After washing, blots were incubated with secondary antibody for 1 h, and X-ray sheets were developed using Immobilon Western Chemiluminescent HRP. Western blot images were quantified using ImageJ software (64-bit Java 1.8.0_172 Wayne Rasband, Research Services Branch, National Institute of Mental Health, Bethesda, MD, USA).

Isolation and quantification of sphingolipids using LC-MS/MS. Collection of cell pellets, lipid isolation, LC-MS/MS analysis, and absolute quantitation of sphingolipids was performed as per our previously published method [32].

Animal studies. Animal experimental protocols were reviewed and approved by the Institutional Animal Ethics Committee of Regional Centre for Biotechnology (RCB/IAEC/2021/96). The experiments were carried out as per the guidelines issued by Committee for the Purpose of Control and Supervision of Experiments on Animals (CPCSEA), Govt. of India.

All tumor growth kinetic experiments were performed in female NOD SCID CB.17 mice. Prior to the cell injection, hair was shaved from flank of the mice. For comparative tumor kinetics experiments, respective cell lines (BT-474 and MDA-MB-231) were resuspended in FBS: Matrigel (1:1, 200 µL), and 3 × 10^6^ cells were injected subcutaneously in the flank. Once the tumor was palpable (20–30 mm^3^), tumor volume and weight of the mice were recorded after every two days. Tumor volume was calculated as per the formula, L × B^2^/2 where L is the length of tumor and B is breadth of tumor. At the end of the experiment, tumors were excised, washed with ice-cold 1X PBS, and stored in Allprotect tissue reagent at −80 °C for further analysis. 

For siRNA experiments, mice with palpable tumor (20–30 mm^3^) were randomized into different groups with each group having 4–6 mice. Group 1 mice were left untreated, group 2 mice were injected intratumorally with scrambled siRNA, and group 3 mice were treated with target siRNA. The siRNA was injected in a volume of 40 µL with 300 ng of siRNA complexed with TAC6 polymer (siRNA:TAC6, 1:10) at the tumor site [33]. A total of 6 doses were given, and tumor measurements were performed at an interval of every two days. At the end of experiment, tumors were excised and stored in Allprotect tissue reagent at −80 °C for further analysis.

For inhibitor experiments, mice with palpable tumor (20–30 mm^3^) were randomized into different groups with each group having 4–6 mice. Group 1 mice were left untreated, and group 2 mice were implanted subcutaneously with inhibitor (NVP-231)-loaded hydrogel [34,35]. For inhibitor loading a dosage of 20 mg/kg of NVP-231 was entrapped in 70 mg/mL of hydrogel. After every two days, tumor was measured for the two groups.

Visualization of sphingolipid metabolic pathways. Expression of genes involved in sphingolipid metabolism were quantified using RT-PCR. Protein levels were probed by Western blots and quantified using ImageJ, followed by normalization against actin. Lipidomics experiments were performed to quantify the metabolite (sphingolipid) levels. Log2 (fold changes) were calculated for MDA-MB-231 against BT-474 cells. The visualization was made using drawio, a cross-platform JavaScript based open source drawing application available on GitHub at https://github.com/jgraph/drawio-desktop (accessed on 28 February 2021).

## 3. Results

### 3.1. TPBC and TNBC Cell Lines Possess Unique Sphingolipid Profile

We selected BT-474 and MDA-MB-231 breast cancer cell lines representing TPBC and TNBC subtypes. To quantify the levels of sphingolipid species in these two cell lines, we collected cell pellets, and isolated the total lipids enriched in sphingolipids. We performed qualitative and quantitative sphingolipidomics with absolute quantitation of 27 sphingolipid species including ceramides, glucosylceramides, lactosylceramides, sphingomyelins, ceramide-1-phosphates (C1P), sphingosine, and sphingosine-1-phosphate (S1P) using LC-MS/MS [31], and compared the profile of sphingolipids from both cell lines (Figure S1A). A heat map showed higher levels of ceramides, lactosylceramides, C1P, and S1P in MDA-MB-231 cells as compared to BT-474 cells (Figure S1A). In contrast, glucosylceramides and sphingomyelin levels were higher in BT-474 cells in comparison to MDA-MB-231 cells (Figure S1A). 

**Figure 2:**
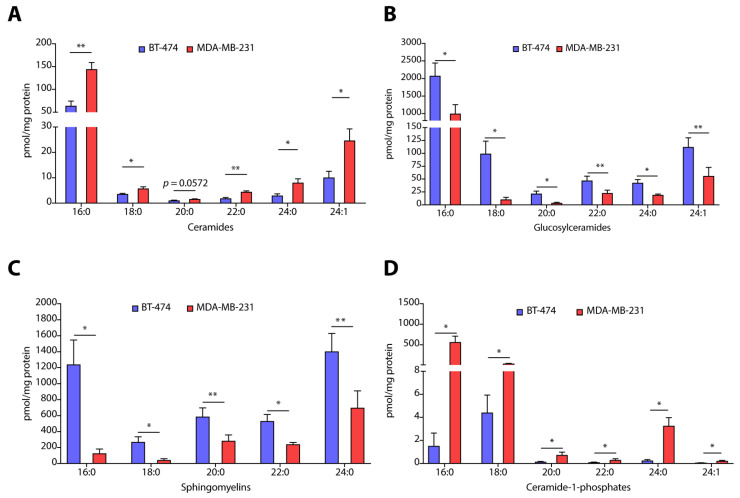
(**A**–**D**) Absolute quantification of different sphingolipids such as ceramides (**A**), glucosylceramides (**B**), sphingomyelins (**C**), and ceramide-1-phosphates (**D**) in TPBC representing BT-474 and TNBC representing MDA-MB-231 cells. Data are presented as mean ± SEM of six independent replicates, and were analyzed by unpaired student’s *t*-test. *p* value: * *p* < 0.05, ** *p* < 0.01.

Absolute quantification of each ceramide species revealed that all species of ceramides including C16:0 (>2.0-fold, *p* < 0.01), C18:0 (>0.5-fold, *p* < 0.05), C22:0 (>2.4-fold, *p* < 0.01), C24:0 (>2.5-fold, *p* < 0.05), and C24:1 (>2.4-fold, *p* < 0.05) were elevated in MDA-MB-231 cells in comparison to BT-474 cells (Figure 2A). Interestingly, all species of glucosylceramides, such as C16:0 (~2.0-fold, *p* < 0.05), C18:0 (~10-fold, *p* < 0.05), C20:0 (>6.0-fold, *p* < 0.05), C22:0 (~4.0-fold, *p* < 0.01), C24:0 (~2-fold, *p* < 0.05), and C24:1 (~2-fold, *p* < 0.01), are downregulated in MDA-MB-231 cells as compared to BT-474 cells (Figure 2B). However, lactosylceramides are more abundant in MDA-MB-231 cells as recorded in the case of C16:0 (>2.7-fold, *p* < 0.05) (Figure S1B) and C24:0 (>6.0-fold, *p* < 0.05) species as compared to BT-474 cells (Figure S1C). We also observed that sphingomyelin species, such as C16:0 (~10-fold, *p* < 0.05), C18:0 (>6.5-fold, *p* < 0.05), C20:0 (~4-fold, *p* = 0.01), C22:0 (~2-fold, *p* < 0.05), and C24:0 (~4-fold, *p* < 0.01), are less abundant in MDA-MB-231 cells as compared to BT-474 cells (Figure 2C). It is interesting to note that all species of C1P including C16:0 (>370-fold, *p* < 0.05), C18:0 (~8.0-fold, *p* < 0.05), C20:0 (>5.5-fold, *p* < 0.05), C22:0 (>3-fold, *p* < 0.05), C24:0 (>13-fold, *p* < 0.05) and C24:1 (>4-fold, *p* < 0.05), are elevated in MDA-MB-231 cells (Figure 2D). We also observed a ~10.0-fold (*p* < 0.01) increase in levels of S1P (Figure S1D) in MDA-MB-231 cells without any change in sphingosine levels (Figure S1E). Therefore, these results reveal that BT-474 and MDA-MB-231 cells representing TPBC and TNBC possess a unique sphingolipid profile that might be responsible for their unique phenotypic characters.

### 3.2. TPBC and TNBC Cells Exhibit Distinct Expression of Sphingolipid-Metabolism Genes

**Figure 3:**
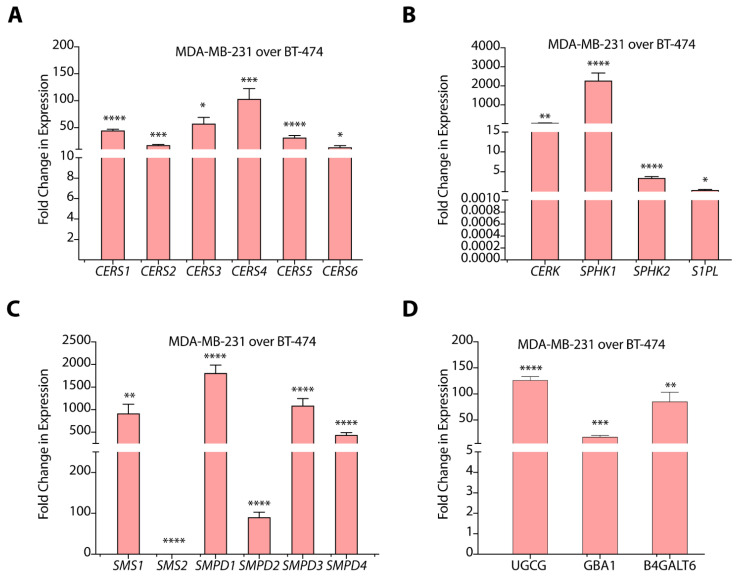
(**A**–**D**) Gene expression data of different sphingolipid-metabolizing genes in MDA-MB-231 cells as compared to BT-474 cells. Data are presented as mean ± SEM of three independent replicates, and were analyzed by unpaired student’s *t*-test. *p* value: * *p* < 0.05, ** *p* < 0.01, *** *p* < 0.001, **** *p* < 0.0001.

Breast cancer subtypes with different histopathological features exhibit varying degrees of heterogeneity and distinct gene expression patterns. Therefore, we evaluated the transcript abundance of sphingolipid-metabolizing genes coding for enzymes of de novo, salvage, and sphingomyelin hydrolysis pathways. We observed that for sphingolipid-metabolizing genes of de novo pathway, MDA-MB-231 cells showed enhanced expression of serine palmitoyl transferase 1 (*SPT1)* (~17-fold, *p* < 0.01), *SPT2* (~6-fold, *p* < 0.001), delta 4-desaturase, sphingolipid 1 and 2 (*DEGS1*) (>6.0-fold, *p* < 0.0001), *DEGS2* (>2.5-fold, *p* < 0.05), and 3-ketodihydrosphingosine reductase (*KDSR*) (>2.0-fold, *p* < 0.01) as compared to BT-474 cells (Figure S2A). We also observed elevated expression of ceramide synthases *CERS1* (>44-fold, *p* < 0.0001), *CERS2* (>17.0-fold, *p* < 0.0001), *CERS3* (>57.0-fold, *p* < 0.001), *CERS4* (>103-fold, *p* < 0.0005), *CERS5* (>31.0-fold, *p* < 0.0001), and *CERS6* (>13.0-fold, *p* < 0.01) in MDA-MB-231 cells as compared to BT-474 cells (Figure 3A). Therefore, these results suggest that de novo biosynthetic pathway of ceramide synthesis is significantly upregulated in TNBC cells as compared to TPBC cells.

Among salvage pathway genes, expression of *N*-Acylsphingosine amidohydrolase 1 and 2 *ASAH1* (>13.0-fold, *p* < 0.05), *ASAH2* (>3.0-fold, *p* < 0.05), alkaline ceramidase 1 and 3 *ACER1* (>518-fold, *p* < 0.05), and *ACER3* (~3.0-fold, *p* < 0.0001) are significantly upregulated in MDA-MB-231 cells as compared to BT-474 cells (Figure S2B). This increase in the expression of ceramidases might be responsible for enhanced S1P levels in MDA-MB-231 cells. MDA-MB-231 cells also showed an abundant and significant increase (*p* < 0.0005) in the expression of sphingosine kinase 1 (*SPHK1*) and a ~3.3-fold (*p* < 0.0005) increase in *SPHK2* as compared to BT-474 cells that, again, positively correlates to higher S1P levels (Figure 3B). Further, we observed that *CERK* expression is upregulated transcriptionally (>45.0-fold, *p* < 0.01) in MDA-MB-231 cells (Figure 3B) that correlates well with higher C1P levels. Further, we observed a >2.5-fold (*p* < 0.01) decrease in the expression of S1P lyase (*S1PL*) in MDA-MB-231 cells over BT-474 cells.

In the sphingomyelin hydrolysis pathway, we observed high expression of sphingomyelin phosphodiesterase 1 (*SMPD1*) (*p* < 0.0001), *SMPD2* (>88.0-fold, *p* < 0.0001), *SMPD3* (>1000.0-fold, *p* < 0.0001), and *SMPD4* (>400.0-fold, *p* < 0.0001) in MDA-MB-231 cells as compared to BT-474 cells (Figure 3C). The gene expression pattern for SMPDs support higher levels of sphingomyelins in BT-474 cells as compared to MDA-MB-231 cells. Interestingly, we observed elevated expression of sphingomyelin synthase 1 (*SMS1)* (*p* < 0.0001), and downregulation of SMS2 (*p* < 0.0001) in MDA-MB-231 cells (Figure 3C). High expression of SMPDs correlated to high ceramide levels in MDA-MB-231 cells suggest efficient sphingomyelin hydrolysis in spite of high *SMS1* expression.

We observed a >100.0-fold increase (*p* < 0.0001) in glucosylceramide synthase (*UGCG*) and a >50-fold increase (*p* < 0.01) in the expression of *B4GALT6* in MDA-MB-231 cells in comparison to BT-474 cells (Figure 3D). In spite of high *UGCG* expression, MDA-MB-231 cells showed lower glucosylceramide levels that might be due to their conversion to lactosylceramides due to the upregulation of *B4GALT6*. MDA-MB-231 cells also showed a ~17-fold increase (*p* < 0.0005) in glucosylceramidase 1 (*GBA1*) expression, responsible for breakdown of glucosylceramides, but we could not detect galactosidase (*GLB1*) expression due to low transcript levels. High expression of *B4GALT6*, thereby, support high lactosylceramide levels in MDA-MB-231 cells as compared to BT-474 cells in spite of high *GBA1* expression (Figure 3D).

### 3.3. TPBC and TNBC Cells Exhibit Distinct Expression of Sphingolipid-Metabolizing Enzymes

**Figure 4:**
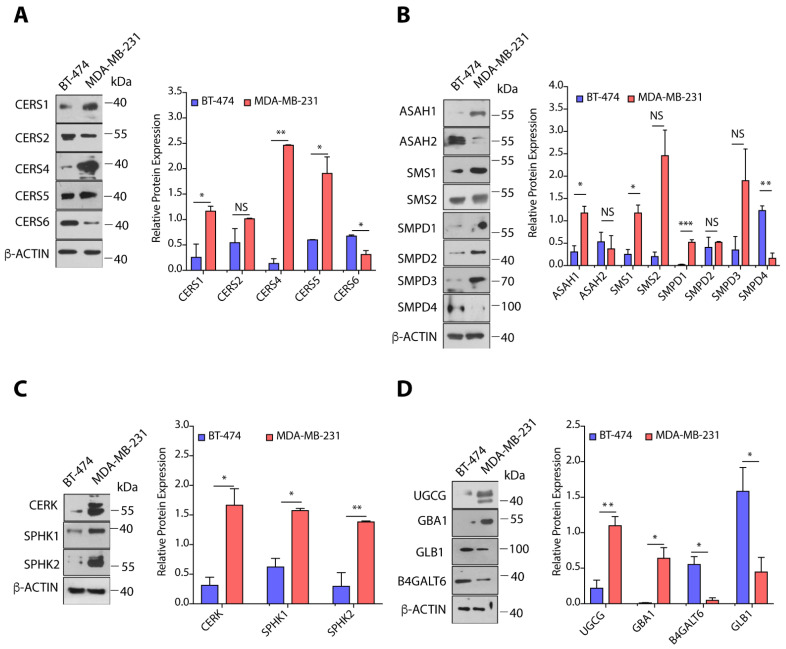
(**A**–**D**) Immunoblots and quantification of different proteins of sphingolipid metabolic pathway in BT-474 and MDA-MB-231 cells along with representative *β*actin as control. Protein expression was normalized with representative *β*-actin as control. Details of all Immunoblots with originals are provided in Figure S4. Data are presented as mean ± SEM of at least three independent replicates, and were analyzed by unpaired student’s *t*-test. *p*-value: * *p* < 0.05, ** *p* < 0.01, *** *p* < 0.001.

We selected some of the sphingolipid-metabolizing enzymes from our gene expression results, and compared the protein expression of these enzymes in BT-474 and MDA-MB-231 cells by immunoblotting. Fold-change was calculated by dividing the protein expression in MDA-MB-231 cells over BT-474 cells, and expression in BT-474 cells was taken as 1. MDA-MB-231 cells showed multi-fold increase in the expression of CERS1 (>4.0-fold, *p* < 0.05), CERS4 (>17.0-fold, *p* < 0.01), CERS5 (>3.0-fold, *p* < 0.05), but there was >2.0-fold (*p* < 0.05) decrease in the expression of CERS6 in comparison to BT-474 cells (Figure 4A). Increase in the expression of CERS1, CERS4, and CERS5 correlated very well with changes in the gene expression of corresponding transcripts and levels of ceramides. Interestingly, though we observed high *CERS6* (>9.0-fold, *p* < 0.0001) gene expression in MDA-MB-231 cells as compared to BT-474 cells but it is not translated to protein expression, that might be due to different post-transcriptional and post-translational regulatory changes (Figure 4A). High expression of SMPD1 (~20.0-fold, *p* < 0.001) in MDA-MB-231 as compared to BT-474 cells is very well correlated with gene expression data and lower levels of sphingomyelins in MDA-MB-231 cells (Figure 4B). Although MDA-MB-231 cells showed high expression of SMS1 (>4.0-fold, *p* < 0.05), SMS2 (>12.0-fold, *p* = 0.059) and lower levels of SMPD4 (>7.0-fold, *p* < 0.01) as compared to BT-474 cells (Figure 4B), it is the overall balance between SMS1, SMS2, and SMPD1-4 that drives higher levels of sphingomyelins in BT-474 cells and increased ceramide levels in MDA-MB-231 cells. 

MDA-MB-231 cells also showed a >3.5-fold (*p* < 0.05) increase in the expression of ASAH1 as expected from gene expression data, whereas there was no significant change in ASAH2 protein expression (Figure 4B). As MDA-MB-231 cells showed transcriptional upregulation of CERK, SPHK1, and SPHK2, we also observed enhanced expression of CERK (>5.0-fold, *p* < 0.05), SPHK1 (>2.5-fold, *p* < 0.05), and SPHK2 (>4.5-fold, *p* < 0.01) proteins in MDA-MB-231 cells as compared to BT-474 cells (Figure 4C). Therefore, this upregulated expression of ASAH1, CERK, SPHK1, and SPHK2 is responsible for increased levels of C1P and S1P in MDA-MB-231 cells as compared to BT-474 cells. As observed in gene expression results, we observed a ~5.0-fold (*p* < 0.01) increase in UGCG expression in MDA-MB-231 cells in comparison to BT-474 cells, but there was a >7.5-fold decrease (*p* < 0.05) in the expression of B4GALT6 (Figure 4D). Interestingly, we observed high levels of GBA1 (>36.0-fold, *p* < 0.05) and low expression of GLB1 (~5.0-fold, *p* < 0.05) in MDA-MB-231 cells as compared to BT-474 cells (Figure 4D). It is plausible that the dynamic equilibrium between ceramides, glucosylceramides, and lactosylceramides is shifted towards high lactosylceramides in MDA-MB-231 cells through combined effect of UGCG, GBA1, B4GALT6, and GLB1 expression. 

### 3.4. TPBC and TNBC Cell Lines Show Differential Cell Proliferation and Migration Abilities

**Figure 5:**
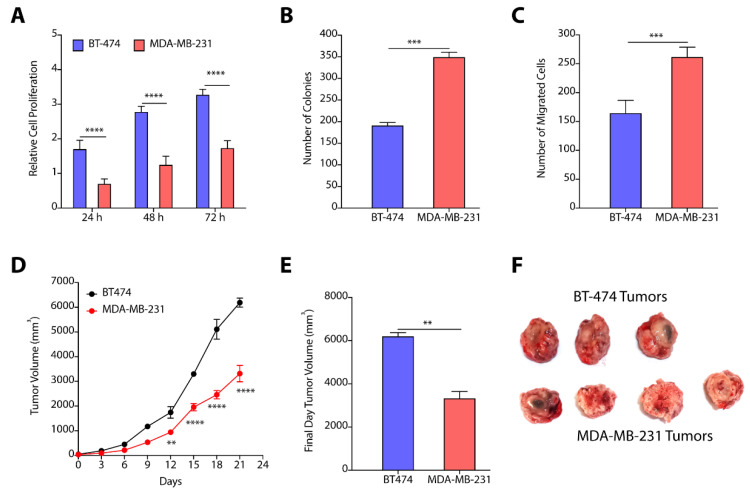
(**A**) Relative cell proliferation kinetics of BT-474 and MDA-MB-231 cells show enhanced proliferation of BT-474 cells as compared to MDA-MB-231 cells. (**B**) Number of colonies formed by BT-474 and MDA-MB-231 cells recorded higher number of colonies formed by MDA-MB-231 cells. (**C**) Quantification of number of migrated cells reveal higher number of MDA-MB-231 cells as compared to BT-474 cells. (**D**–**F**) Tumor growth kinetics (**D**), final day tumor volume (**E**), and pictures of excised tumors (**F**) witness enhanced proliferation of BT-474 tumors. Data in (**A**–**C**) are presented as mean ± SEM of at least three independent replicates, and were analyzed by unpaired student’s *t*-test for. Data in (**D**,**E**) are presented as mean ± SEM (*n* = 3/4), and were analyzed using two-way ANOVA for Figure 5(**D**), and using unpaired student’s *t*-test for (**E**). *p*-value: ** *p* < 0.01, *** *p* < 0.001, **** *p* < 0.0001.

Next, we determined the cell proliferation rates of BT-474 and MDA-MB-231 cells where cells (~5000 cells/well) were plated in 96-well plate, and proliferation index was determined by MTT assay. We observed that BT-474 cells exhibit >2.5-fold higher (*p* < 0.0001) proliferation at 72 h as compared to MDA-MB-231 cells (Figure 5A). Colony forming assay showed that MDA-MB-231 have higher tendency to form colonies as compared to BT-474 cells (Figure 5B). To compare the invasion ability of cells, we performed transwell migration assay, and observed a ~1.3-fold (*p* < 0.001) increase in migration of MDA-MB-231 cells as compared to BT-474 cells (Figure 5C). We next determined the tumor growth kinetics of BT-474 and MDA-MB-231 cells. We implanted 3.0 million cells in NOD-SCID mice, and measured the tumor growth kinetics. BT-474 tumors have significantly higher proliferation rate than MDA-MB-231 tumors (Figure 5D), and showed a ~2-fold increase in volume on final day as compared to MDA-MB-231 tumors (Figure 5E,F).

Our data from proliferation, colony forming, and migration assays confirmed that BT-474 cells exhibit higher cell proliferation rate as compared to MDA-MB-231 cells, and MDA-MB-231 cells demonstrate higher migration rate that makes the two subtype-specific cell lines unique in their phenotypic behavior. Next, we attempted to correlate the sphingolipid levels and gene/protein expression of sphingolipid-metabolizing enzymes from BT-474 and MDA-MB-231 cells with their cell proliferation and migration properties to show how these two subtype-specific cells modulate the sphingolipid metabolism to elicit their distinct responses. We identified that increased levels of ceramides due to enhanced expression of ceramide synthases in MDA-MB-231 cells might be responsible for their lower proliferation rate as compared to BT-474 cells. Similarly, increased levels of sphingomyelins might be responsible for the high proliferation of BT-474 cells as compared to MDA-MB-231 cells. Although, MDA-MB-231 cells have high levels of SMS1 but they also express high levels of SMPDs responsible for the degradation of sphingomyelins. Interestingly, MDA-MB-231 cells show enhanced expression of CERK, SPHK1, and SPHK2 corelating with high levels of C1P and S1P that might be responsible for their higher migration abilities. As CERK plays a dual role, in migration and cell proliferation [15,16,17], we hypothesized that CERK can be explored as a common therapeutic target to mitigate cell proliferation and migration in BT-474 and MDA-MB-231 cells.

### 3.5. CERK Inhibition Reduces Cell Proliferation, Colony Formation, and Migration in TPBC and TNBC Cells

**Figure 6:**
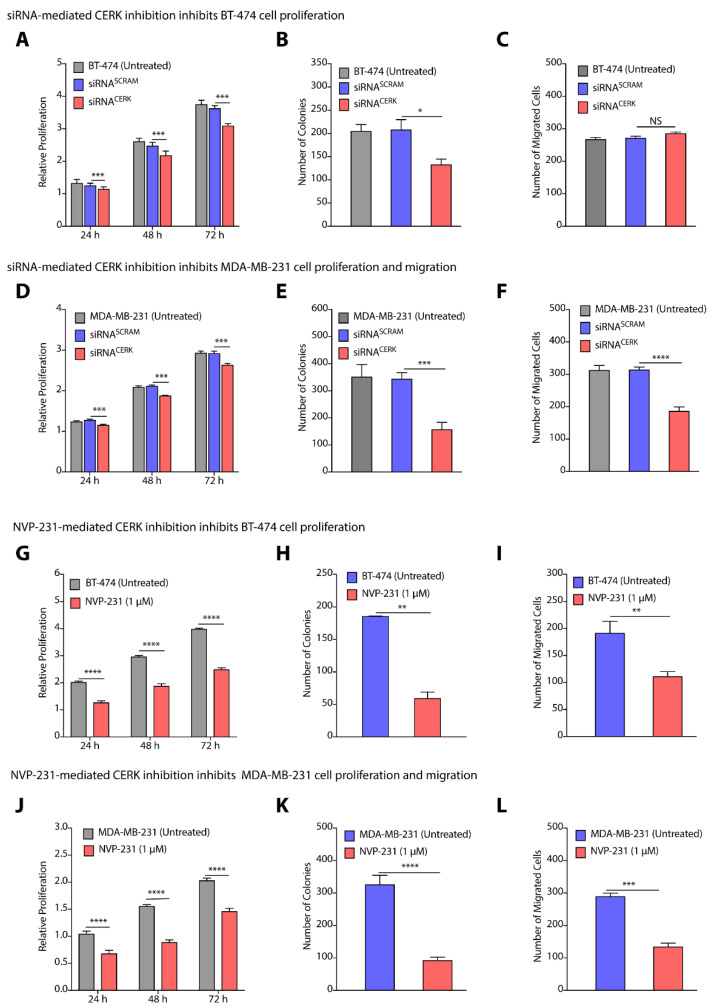
(**A**–**F**) Change in proliferation (**A**,**D**), colony forming units (**B**,**E**), and number of migrated cells (**C**,**F**) on siRNA-mediated knockdown of CERK in BT-474 (**A**–**C**) and MDA-MB-231 (**D**–**F**) cells. (**G**–**L**) Change in proliferation (**G**,**J**), colony forming units (**H**,**K**), and number of migrated cells (**I**,**L**) on NVP-231-mediated inhibition of CERK in BT-474 (**G**–**I**) and MDA-MB-231 (**J**–**L**) cells. Data are presented as mean ± SEM of at least three independent replicates, and were analyzed by unpaired student’s *t*-test. *p*-value: * *p* < 0.05, ** *p* < 0.01, *** *p* < 0.001, **** *p* < 0.0001.

To validate CERK as a common therapeutic target for TPBC and TNBC cells, we used siRNA-mediated knockdown of CERK as well as small molecule-mediated inhibition of CERK, and tested their impact on cell proliferation, colony formation, and cell migration in BT-474 and MDA-MB-231 cells. We observed that siRNA-mediated downregulation of CERK reduced the cell proliferation (>1.13-fold, *p* < 0.005) of BT-474 cells after 72 h (Figure 6A). Knockdown of CERK by siRNA induced a >1.5-fold (*p* < 0.05) decrease in the number of colonies in BT-474 cells (Figure 6B). siRNA-mediated knockdown of CERK did not reflect any significant change in number of migrating BT-474 cells (Figure 6C). Interestingly, there is a significant decrease (>1.2-fold, *p* < 0.001) in cell proliferation (Figure 6D), number of colonies (>3.0-fold, *p* < 0.001) (Figure 6E), and cell migration (~2.0-fold, *p* < 0.0001) on knockdown of CERK by siRNA in MDA-MB-231 cells (Figure 6F).

Next, we used NVP-231, a small molecule that is known to inhibit CERK activity [35], and tested the impact of NVP-231 on proliferation, colony formation, and migration of BT-474 and MDA-MB-231 cells. Treatment of BT-474 cells with NVP-231 at 1.0µM caused a >39.0-fold (*p* < 0.05) decrease in cell proliferation (Figure 6G). NVP-231 (1.0 µM) treatment also caused a >3.0-fold (*p* < 0.01) decrease in number of colonies over untreated cells (Figure 6H). Transwell migration assay witnessed a >1.5-fold decrease in number of migrated cells on NVP-231 (1.0 µM) treatment (Figure 6I). Similarly, we observed a >1.7-fold (*p* < 0.0001) decrease in cell proliferation of MDA-MB-231 cells on treatment with 1.0 µM of NVP-231 (Figure 6J), and there was a >3.0-fold (*p* < 0.0001) decrease in number of colonies (Figure 6K), and a >2.0-fold (*p* < 0.0001) decrease in number of migrated cells (Figure 6L). Therefore, these results clearly validated that CERK can be a common therapeutic target for TPBC and TNBC cells where CERK inhibition at translational level by siRNA and at enzymatic level by chemical inhibitor diminishes proliferation and cell migration in BT-474 and MDA-MB-231 cells.

### 3.6. Nanoparticle-Mediated Localized Delivery of CERK siRNA Inhibits Tumor Progression

Targeting solid tumors using chemotherapeutic and gene therapeutic strategies through systemic route of administration provide poor success due to engulfment of the delivery vehicles by the reticuloendothelial system, and off-target toxicities of the therapeutics [36]. In contrast, localized delivery of chemotherapeutics provides a suitable alternative as it reduces the dose regimens and avoids systemic toxicity [37,38]. Therefore, we used two strategies to evaluate the effect of pharmacological manipulations of CERK on tumor progression. In the first strategy, we used nanoparticle-mediated delivery of siRNA targeting CERK to tumor tissues, and in the second strategy, we used hydrogel-mediated localized delivery of CERK inhibitor near the tumor site, and determined their effect on tumor growth. 

**Figure 7:**
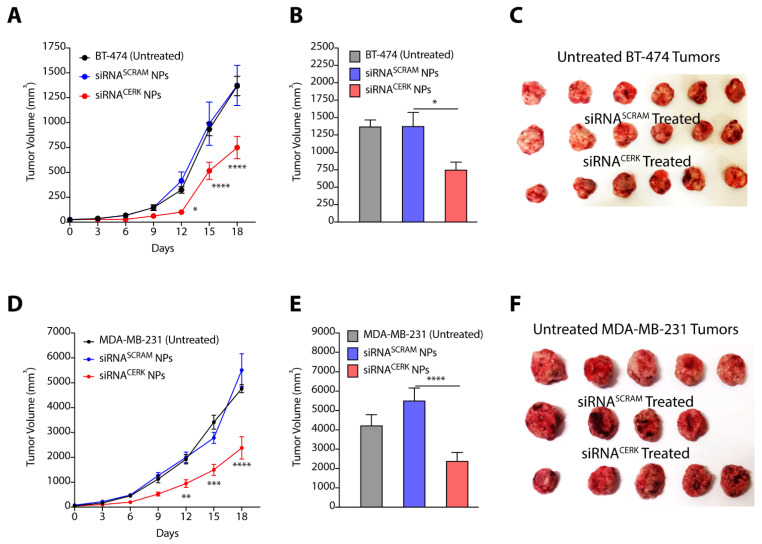
(**A**–**C**) Tumor growth kinetics (**A**), final day tumor volume (**B**), and pictures of excised tumors (**C**) showing the effect of siRNA^CERK^ NPs targeting CERK in BT-474 tumors. (**D**–**F**) Tumor growth kinetics (**D**), final day tumor volume (**E**), and pictures of excised tumors (**F**) showing the effect of siRNA^CERK^ NPs targeting CERK in MDA-MB-231 tumors. Data are presented as mean ± SEM (*n* = 4–6), and were analyzed using two-way ANOVA for (**A**,**D**), and using unpaired student’s *t*-test for (**B**,**E**). * *p* < 0.05, ** *p* < 0.01, *** *p* < 0.001, **** *p* < 0.0001.

In our earlier studies, we have presented the engineering of polyaspartate-derived cationic polymers that can deliver therapeutic siRNA [33]. We therefore used the most effective polymer (TAC6) and engineered complexes of siRNA targeting CERK and TAC6 (called siRNA NPs) at optimized w/w ratio of 10. Next, we randomized the BT-474 and MDA-MB-231 tumor-bearing mice into three groups, where group 1 mice were left untreated, group 2 mice were treated with siRNA^SCRAM^ NPs with scrambled siRNA, and group 3 mice were treated with siRNA^CERK^ NPs with CERK-targeted siRNA. We used six doses of siRNA NPs as per the plan given in Figure S3A. Tumor growth kinetics showed that siRNA-mediated knockdown of CERK inhibited the BT-474 tumor growth as compared to other groups (Figure 7A), and there was a >2.5-fold (*p* < 0.0001) decrease in tumor volume on final day (Figure 7B,C). Similarly, we also observed that NP-mediated delivery of siRNA inhibited growth of MDA-MB-231 tumors (Figure 7D), and there was a >2-fold (*p* < 0.0001) decrease in tumor volume on final day (Figure 7E,F). We validated the knockdown of CERK in tumor tissues by immunoblotting (Figure S3B,C). Therefore, these results confirm that NP-mediated localized delivery of siRNA targeting CERK can be an effective strategy to reduce the tumor growth kinetics in TPBC and TNBC models.

### 3.7. Hydrogel-Mediated Delivery of CERK Inhibitor Mitigates Tumor Progression

**Figure 8:**
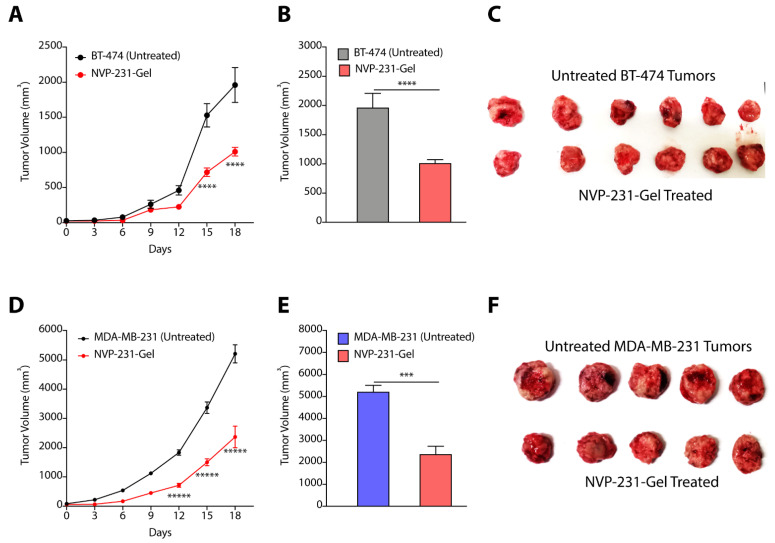
(**A**–**C**) Tumor growth kinetics (**A**), final day tumor volume (**B**), and pictures of excised tumors (**C**) showing the effect of NVP-231-Gel in BT-474 tumors. (**D**–**F**) Tumor growth kinetics (**D**), final day tumor volume (**E**), and pictures of excised tumors (**F**) showing the effect of NVP-231-Gel on MDA-MB-231 tumors. Data are presented as mean ± SEM (*n* = 5/6), and were analyzed using two-way ANOVA for (**A**,**D**), and using unpaired student’s *t*-test for (**B**,**E**). *** *p* < 0.001, **** *p* < 0.0001, ***** *p* < 0.00001.

In our earlier studies, we have presented the synthesis of a glycine-glycine derivative of lithocholic acid that can self-assemble to form supramolecular hydrogel in water [32]. We have shown that the gel can entrap hydrophobic and hydrophilic drug effectively, and can maintain sustained release of chemotherapeutic drugs [37,38]. We, therefore, entrapped NVP-231 in hydrogel where gelator (70 mg) and inhibitor (20 mg) were suspended in water, and heated to form a clear solution. The mixture was then allowed to cool to form hydrogel (called NVP-231-Gel). We then randomized the BT-474 and MDA-MB-231 tumor-bearing mice into two groups where group 1 mice were left untreated, and NVP-231-Gel was subcutaneously implanted near the tumor site in group 2 mice (Figure S3D). Tumor growth kinetic studies clearly demonstrated that treatment of BT-474 tumors with NVP-231-Gel slowed kinetics of tumor growth (Figure 8A), and there was a ~2-fold (*p* < 0.0001) decrease in tumor volume with NVP-231-Gel treatment on the final day as compared to untreated tumors (Figure 8B,C). Similarly, there was significant inhibition in tumor growth kinetics of MDA-MB-231 tumors on treatment with NVP-231-Gel (Figure 8D), and a >2-fold (*p* < 0.05) decrease in volume of MDA-MB-231 tumors on final day (Figure 8E,F). Therefore, these results demonstrated that hydrogel-mediated localized delivery of CERK inhibitors can also be an effective strategy to reduce the tumor growth kinetics of TPBC and TNBC tumors.

## 4. Discussion

**Figure 9:**
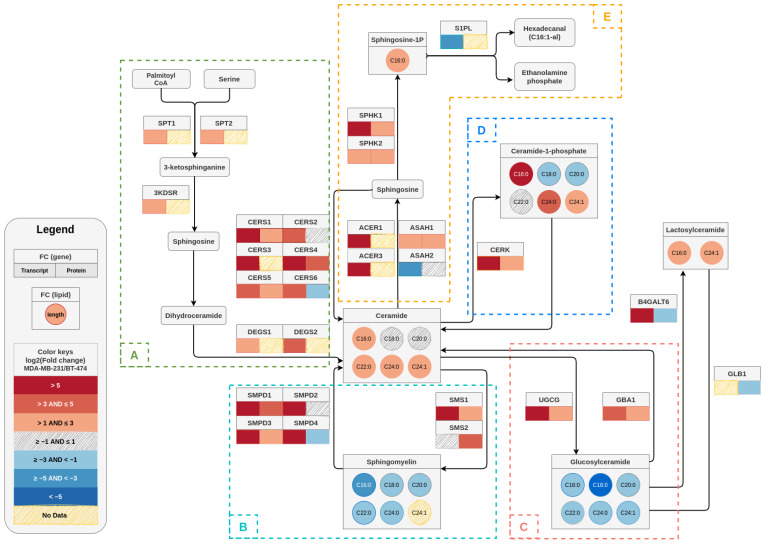
A sphingolipid pathway map depicting the log2 transformed fold changes in transcript, protein, and metabolite (sphingolipid) levels in MDA–MB–231 cells in comparison to BT–474 cells. For each gene, the corresponding transcript and protein fold changes are denoted as color-coded boxes below the gene name (transcript at the left and protein at the right of the box). In case of lipids, circles are used to describe the fold changes for a particular chain length. The color keys given in the legend box describe the color scale of different bins of log2 (fold change) that represents the degree of upregulation or downregulation in gene/protein/metabolites between cell lines The dashed boxes (**A**–**E**) indicate the de novo pathway (**A**) and various salvage pathways (**B**–**E**).

In summary, we elucidated a distinct sphingolipid profile for BT-474 and MDA-MB-231 cells that correlated well with the gene and protein expression pattern for de novo and salvage pathways (Figure 9). We generated a pathway visualization map overlaying the gene/protein expression data and resultant sphingolipid levels for MDA-MB-231 cells in comparison to BT-474 cells [39]. This pathway map pictorially represented the relative differences in levels of sphingolipid metabolites in two subtypes along with alterations in gene and protein expression (fold-change values) of corresponding enzymes (Figure 9). Our results showed that levels of different sphingolipids in TPBC and TNBC cells are tightly regulated and maintained by reversible chemical reactions controlled by specific enzymes, and the net result of these enzymatic regulations dictates the levels of a specific sphingolipid species (Figure 9). Higher ceramide, lactosylceramide, C1P and S1P levels in MDA-MB-231 cells as compared to BT-474 cells could be associated with deregulations in the expression of the genes coding for their respective enzymes [40,41,42]. Attenuated levels of sphingomyelins in MDA-MB-231 cells in spite of high expression of SMS1 and 2 may be attributed to elevated sphingomyelin hydrolysis due to the enhanced expression of SMPDs. Higher glucosylceramide levels in BT-474 did not correlate very well with protein expression levels of the corresponding enzymes. This may be owing to complexities arising from multiple pathways regulating sphingolipid metabolism functioning at the same time so that the flux of metabolites are altered in a more dynamic manner than s envisaged. 

An overview of the gene and sphingometabolic profiles of TPBC and TNBC cells suggest CERK as an apt target to combat tumor cell proliferation and migration in both subtypes. We showed a striking decrease in luminal specific BT-474 cell proliferation and a significant tumor regression in corresponding xenograft model on CERK inhibition. This justified that CERK can potentially be a good therapeutic target for primary tumors of luminal subtype. At the same time, a significant decrease in cell migration in MDA-MB-231 cells and alleviation of growth in the xenograft tumor model implicated that CERK downregulation is equally important for primary tumor abrogation and migration of disseminated tumor cells in TNBC cells. The effect of NVP-231 on CERK activity is more pronounced than siRNA-mediated inhibition of CERK most probably owing to the inhibitory effects of NVP231 on activity of CERK and C1P production as opposed to siRNA-mediated blockage of CERK translation.

CERK expression was found to be higher in ER negative as compared to ER positive breast cancer (luminal subtypes), and worst prognosis, shorter survival and higher recurrence rate were positively associated to higher CERK expression among ER-negative cancers [15,43]. CERK expression was also higher in higher grade tumors independent of molecular subtype. This exhaustive study covering tumor recurrence in genetically engineered mouse models, human breast cancer cell lines, and gene expression profile of 2200 breast cancer patients of all subtypes and clinicopathological characteristics showed a clear correlation of high CERK expression with aggressive tumors and poor clinical outcome following adjuvant or neoadjuvant therapy. The fact that CERK was spontaneously upregulated in different oncogene-derived mammary tumor models rapidly, following withdrawal of chemo/Her2/neu inhibitor therapy strongly indicated the role of CERK/C1P in generation of apoptosis resistant tumor cells that are disseminated from the primary site. Very recently, CERK has also been identified from an inhibitor screen as a potent target to reduce chemotherapy induced senescent cell population that promotes inflammation and reduces the effectiveness of chemotherapy on cancer cells [41]. 

Polymeric and lipid nanoparticles have emerged as effective delivery vehicles for gene therapeutics [44]. In our earlier studies, we showed that oral delivery of gene therapeutics using engineered biodegradable polyaspartate-derived polyplexes can mitigate gut inflammation [33]. Localized delivery of these polyplexes with CERK siRNA reduced the tumor growth kinetics, making it a potential candidate for future gene therapy applications. In recent years, preclinical studies have shown that hydrogel-mediated localized delivery of chemotherapeutics is a promising strategy to mitigate pathophysiology of disease without any toxicity as it does not allow the drugs to be distributed to undesired organs [45,46]. In our earlier studies, we have shown that lithocholic acid-derived hydrogel implant is biodegradable, does not cause any immunogenic response, and is effective in maintaining slow and sustained release of chemotherapeutic drugs at the tumor site [37,38]. Our results showed that this lithocholic acid-derived hydrogel implant can also encapsulate CERK inhibitor, and its implantation near the tumor site can reduce tumor progression. In-depth toxicological and pharmacokinetic studies in large animals of these hydrogel-based implants and polyplexes can provide the potential of these biomaterials for future clinical applications. 

## 5. Conclusions

CERK appears to be playing different roles in more aggressive metastatic breast cancer cell lines versus non-metastatic cell lines targeting cell migration and cell proliferation in a context-dependent manner. Hence, CERK can be a potential target for multiple breast cancer subtypes. However, more studies elucidating the molecular mechanism by which CERK controls these phenotypes and the extent of overlap in the pathways regulating them needs to be performed.

## Data Availability

The data presented in this study are available on request from the corresponding author.

## Supplementary Materials

The following supporting information can be downloaded at: https://www.mdpi.com/article/10.3390/cancers14184496/s1. Figure S1: (A) Heatmap showing the levels of different sphingolipids in BT-474 and MDA-MB-231 cells. (B–E) Absolute quantification of C16:0 lactosylceramides (A), C24:0 lactosylceramides (B), S1P (C), and sphingosine (D) in BT-474 and MDA-MB-231 cells; Figure S2: Gene expression of different sphingolipid-metabolizing genes in MDA-MB-231 cells as compared to BT-474 cells; Figure S3: (A) Schematic showing the experimental design to decipher the effect of siRNA-mediated knockdown of CERK on tumor progression. (B,C) Validation of knockdown of CERK siRNA in BT-474 (B) and MDA-MB-231 (C) tumor tissues by immunoblotting. (D) Schematic showing the experimental design to decipher the effect of NVP-231-Gel-mediated inhibition of CERK on tumor progression. Figure S4: Full Western blot images. Table S1: List of primers (human) used for validation of endogenous gene expression by Real-Time PCR.
