# Supplementary material for: Ceramide Kinase (CERK) Emerges as a Common Therapeutic Target for Triple Positive and Triple Negative Breast Cancer Cells
Source: Cancers (Basel). 2022 Sep 16;14(18):4496. doi: 10.3390/cancers14184496 (PMC9497187; doi:10.3390/cancers14184496)

CERS1

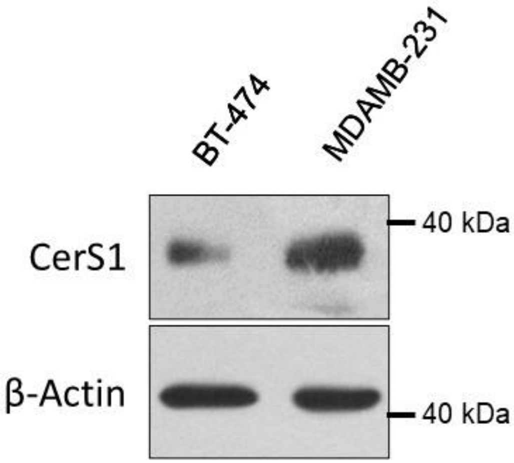

CERS1 Original blot

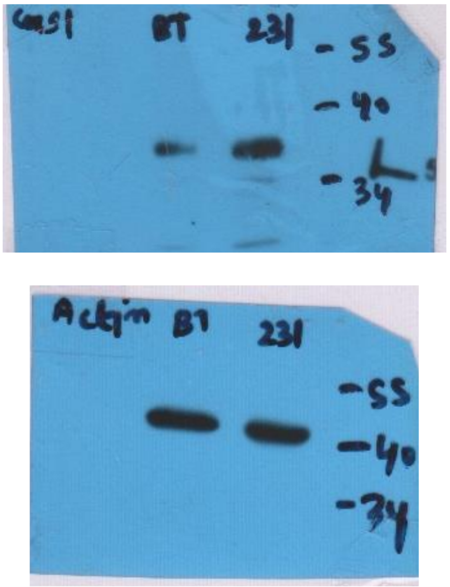

CERS2

Replicate 1

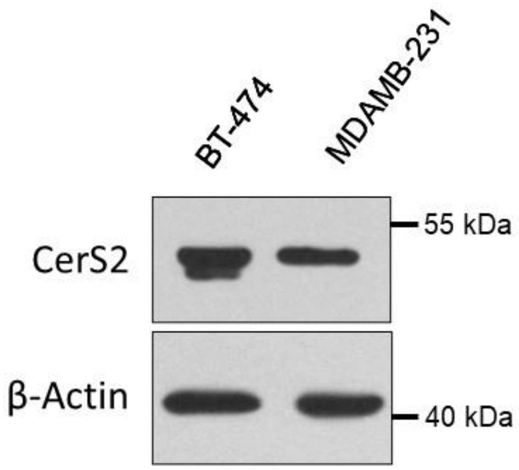

CERS2 Original blot

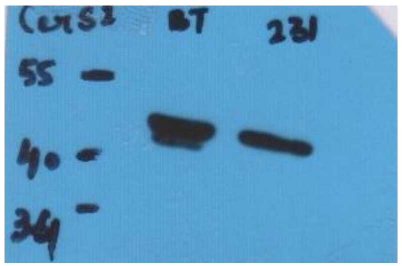

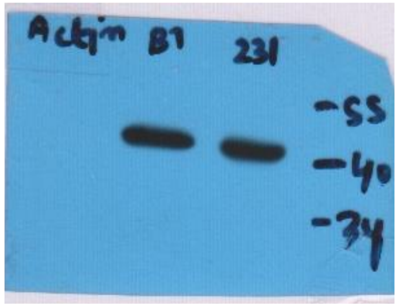

CERS4

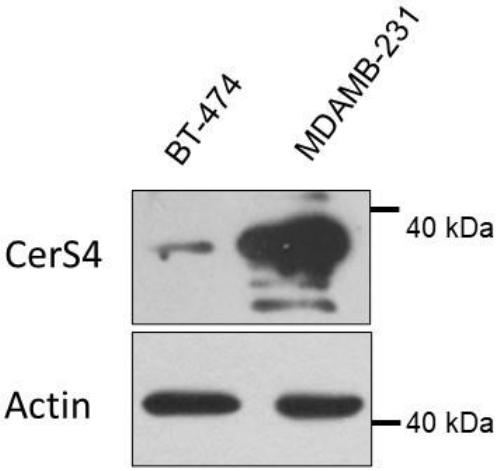

CERS4 Original blot

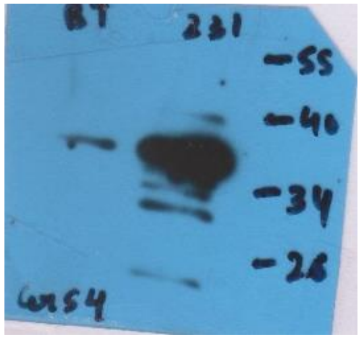

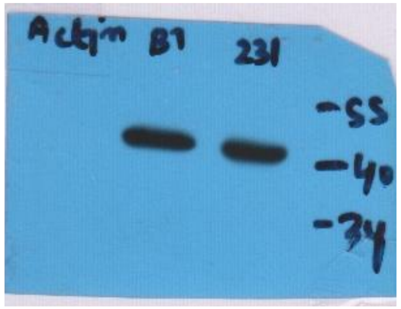

CERS5

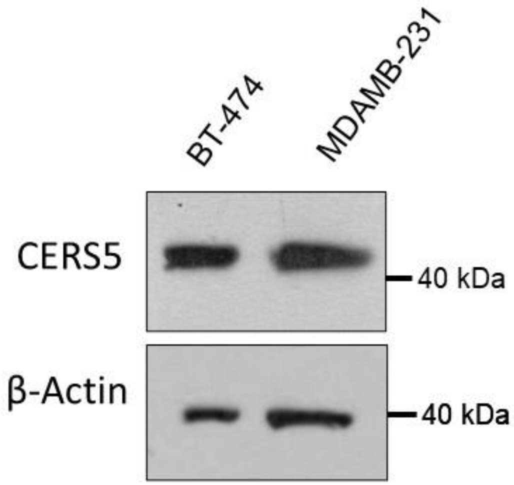

CERS5 Original blot

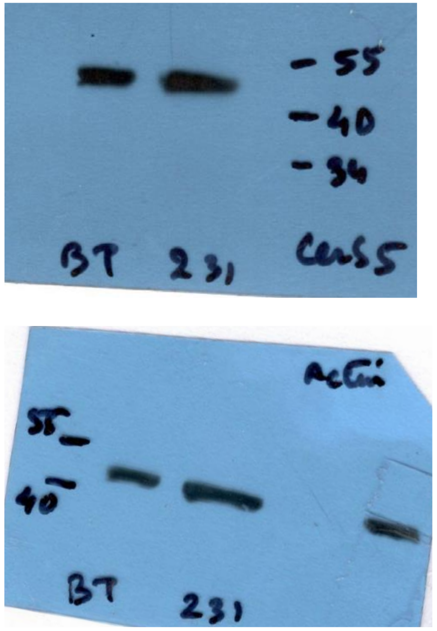

CERS6

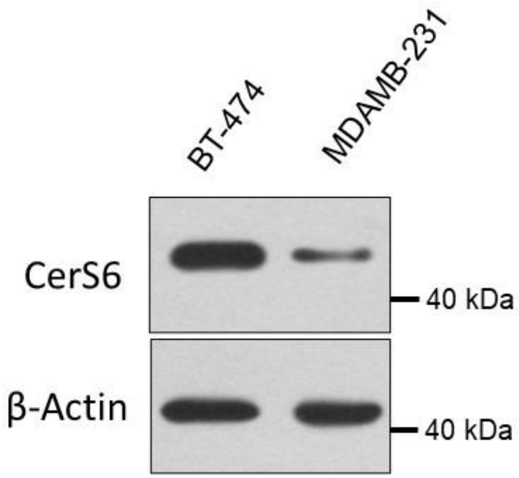

CERS6 Original blot

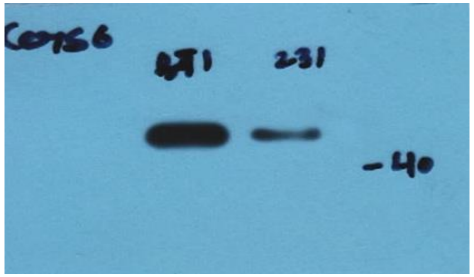

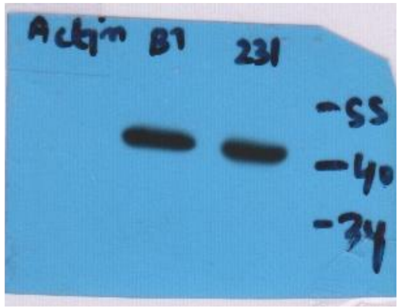

ASAH1

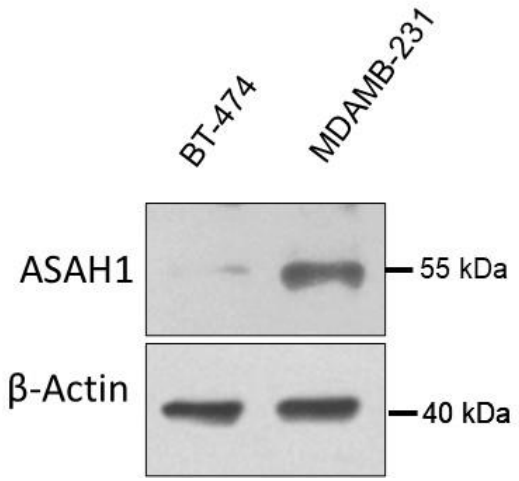

ASAH1 Original blot

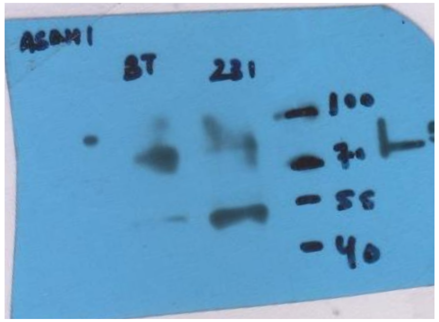

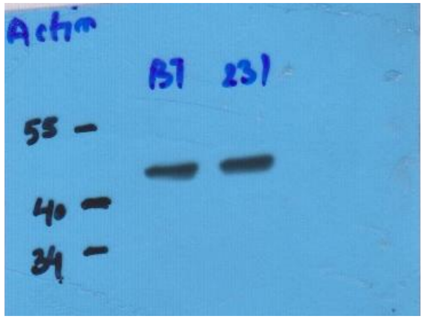

ASAH2

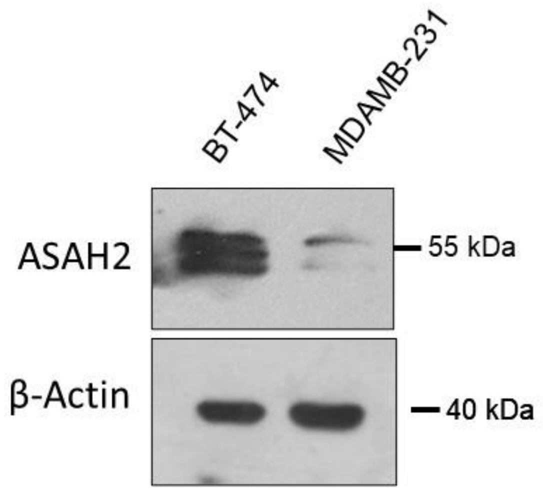

ASAH2 Original blot

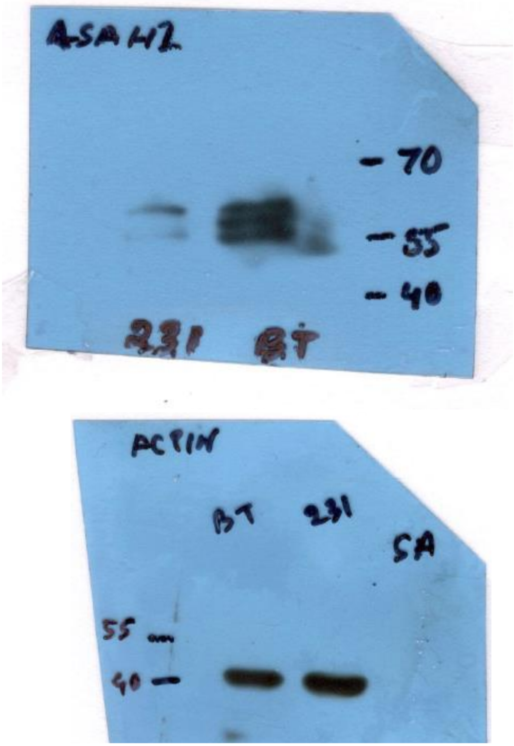

UGCG

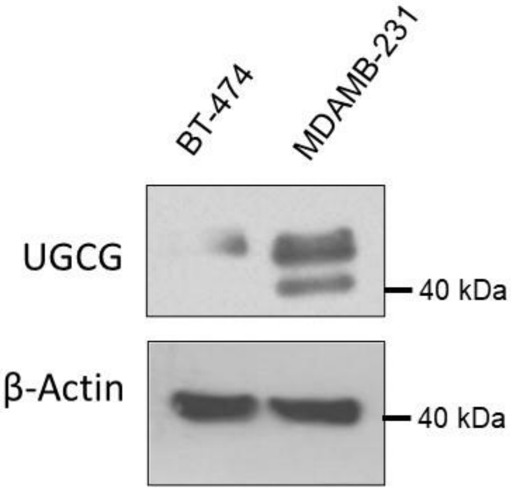

UGCG Original blot

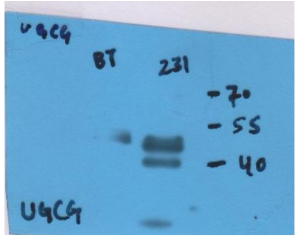

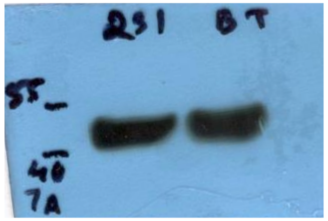

CERK

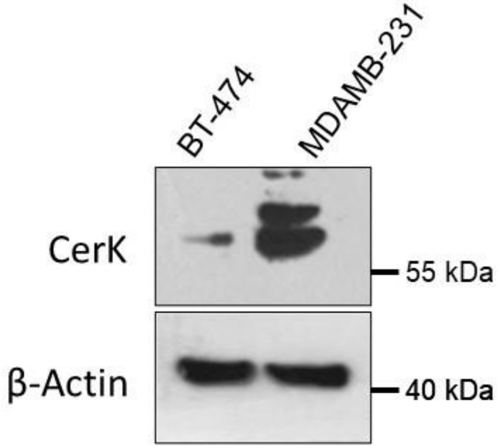

CERK Original blot

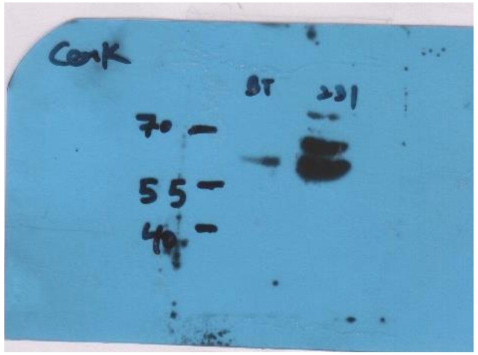

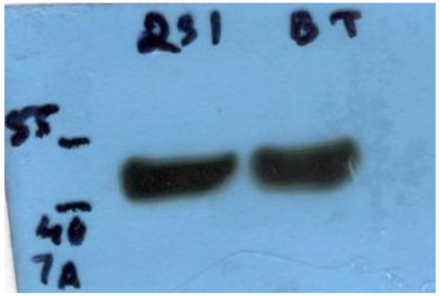

SPHK1

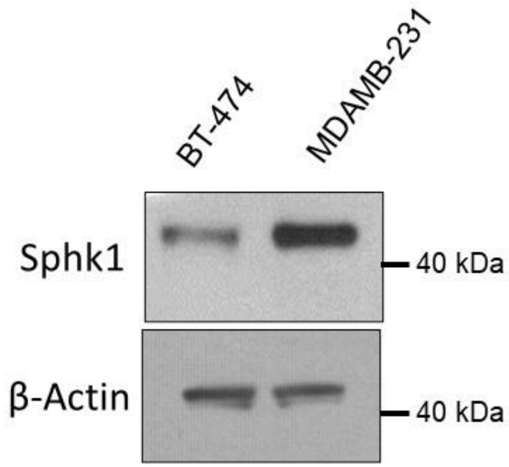

SPHK1Original blot

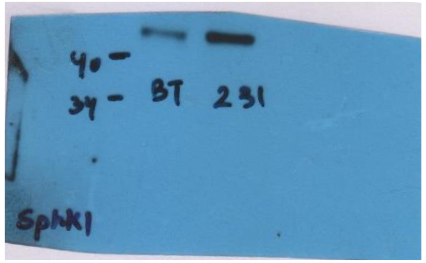

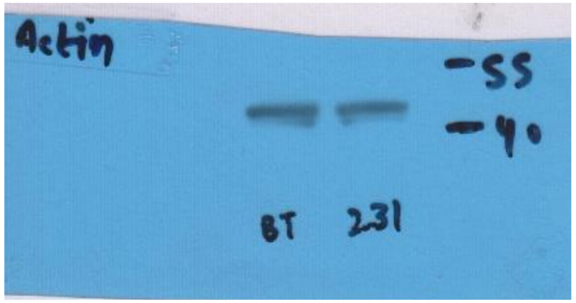

SPHK2

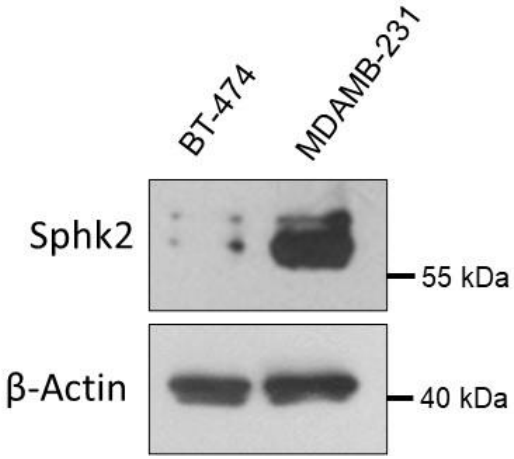

SPHK2 Original blot

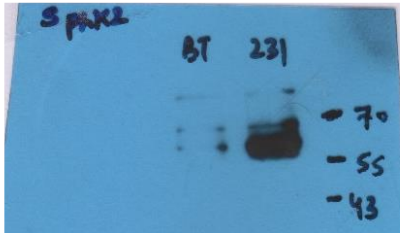

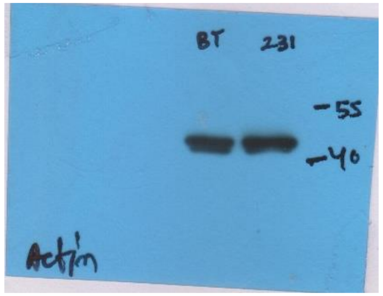

SGMS1

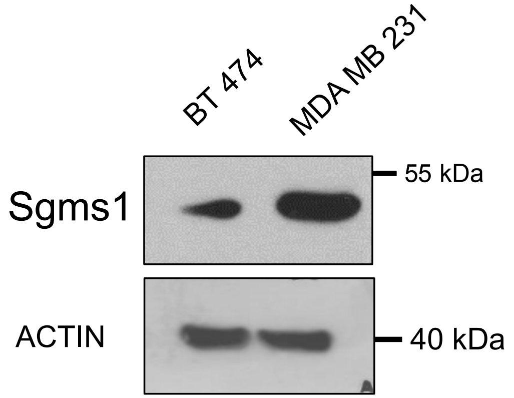

SGMS1 Original blot

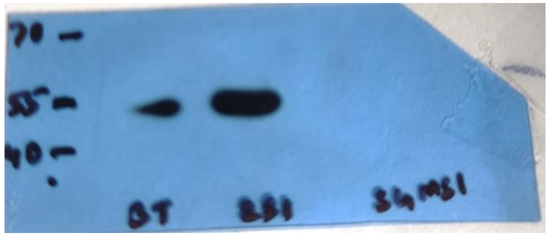

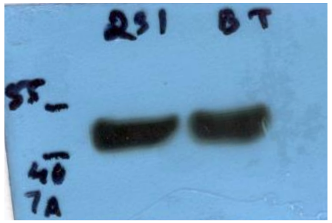

SGMS2

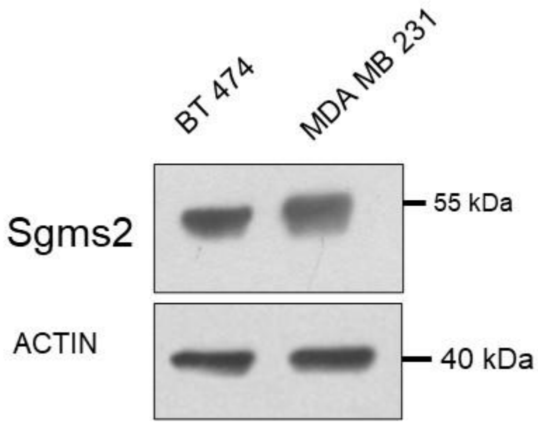

SGMS2 Original blot

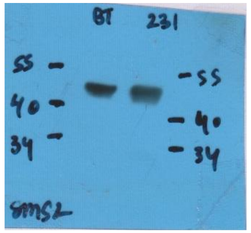

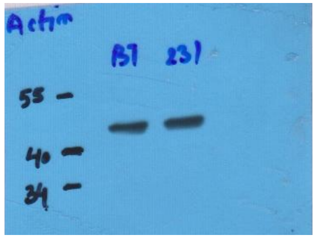

SMPD1

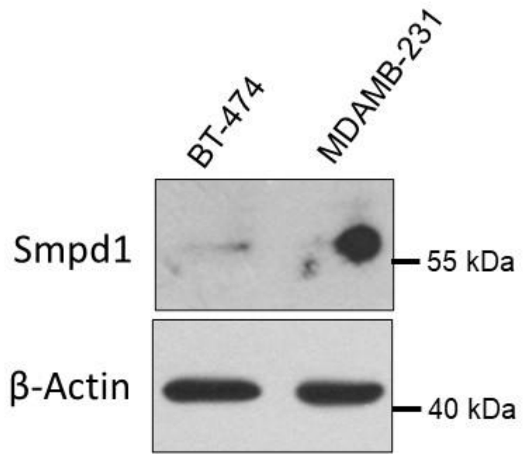

SMPD1 Original blot

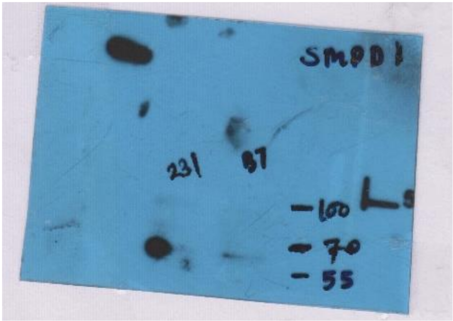

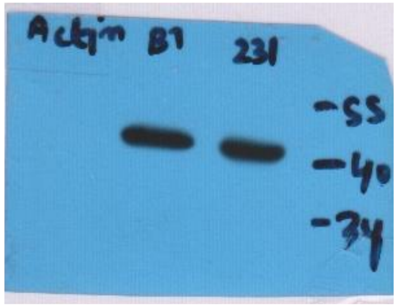

SMPD2

Replicate 1

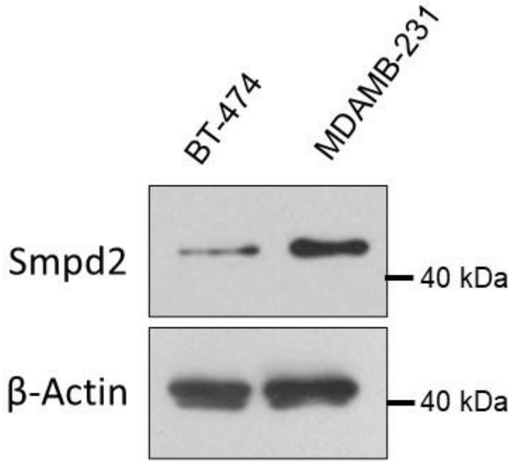

SMPD2 Original blot

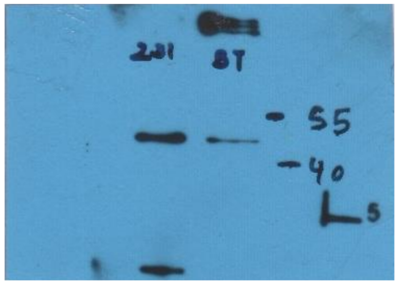

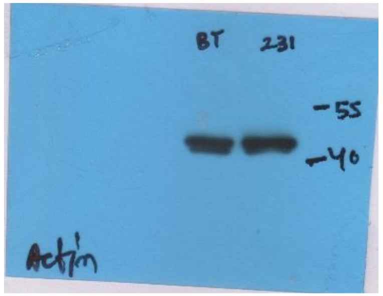

SMPD3

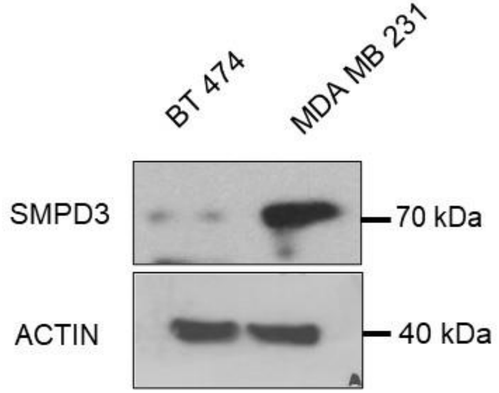

SMPD3 Original blot

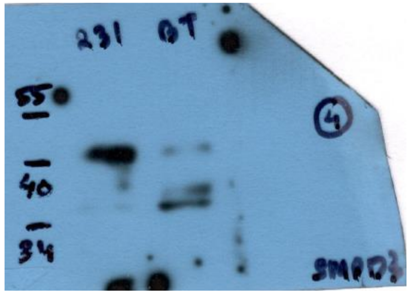

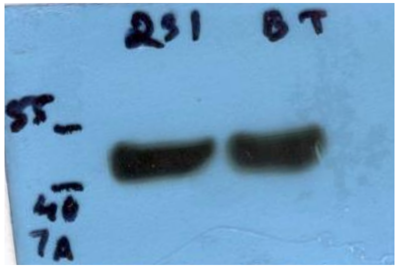

SMPD4

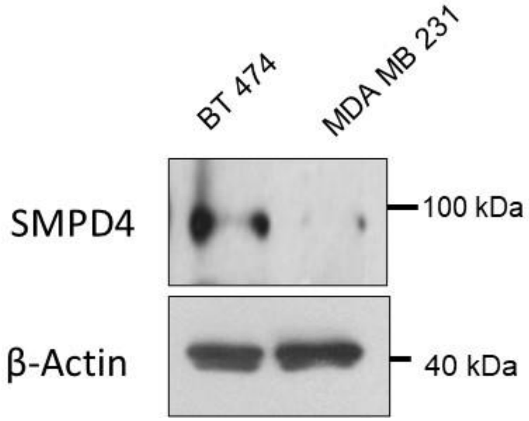

SMPD4 Original blot

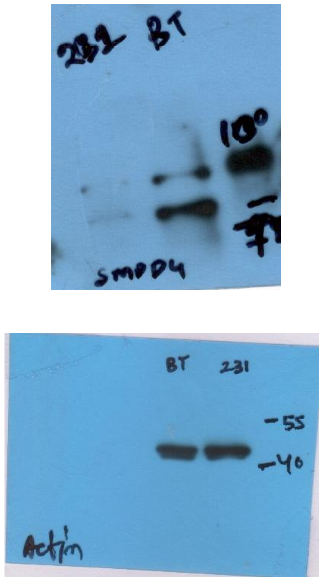

GLB1

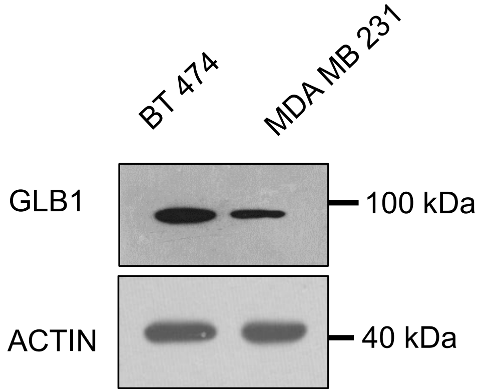

GLB1 Original Blot

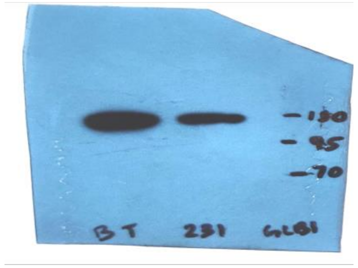

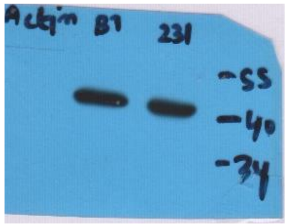

GBA1

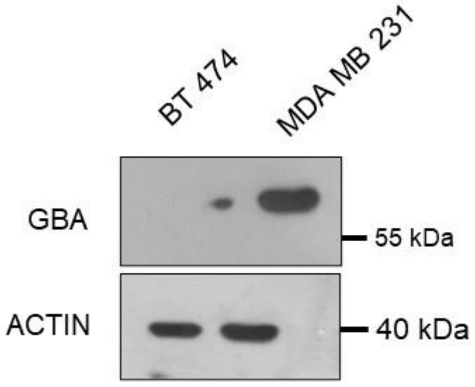

GBA1 Original Blot

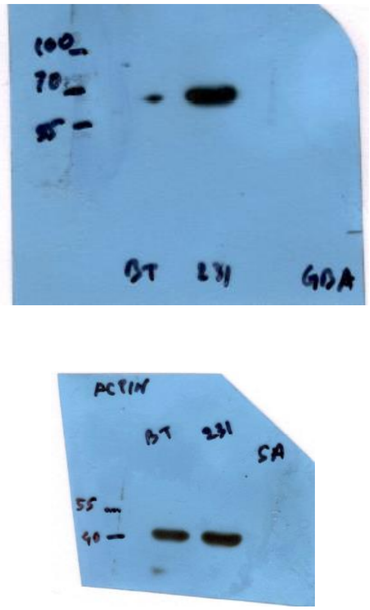

B4GALT6

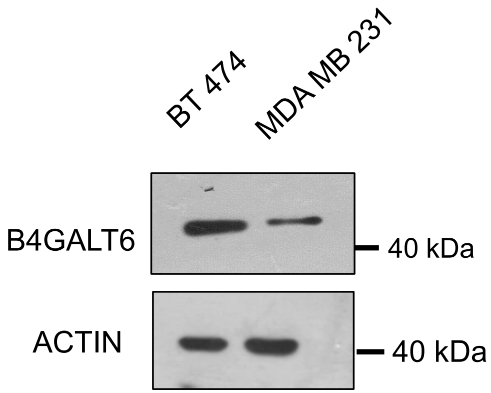

B4GALT6 Original Blot

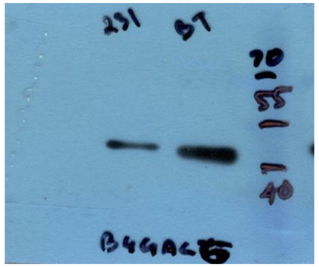

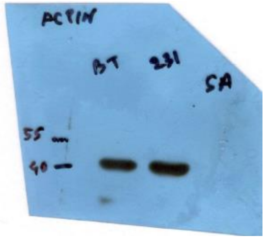

CerK siRNA in BT-474

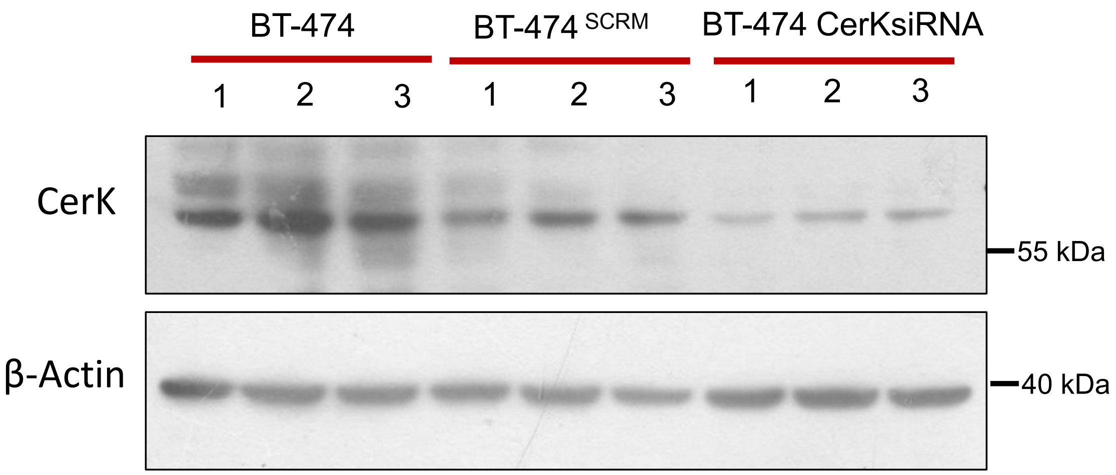

CerK siRNA in BT-474 Original Blot

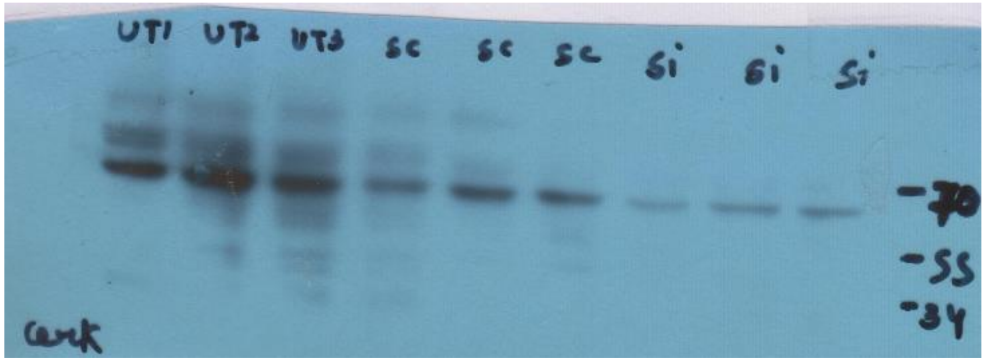

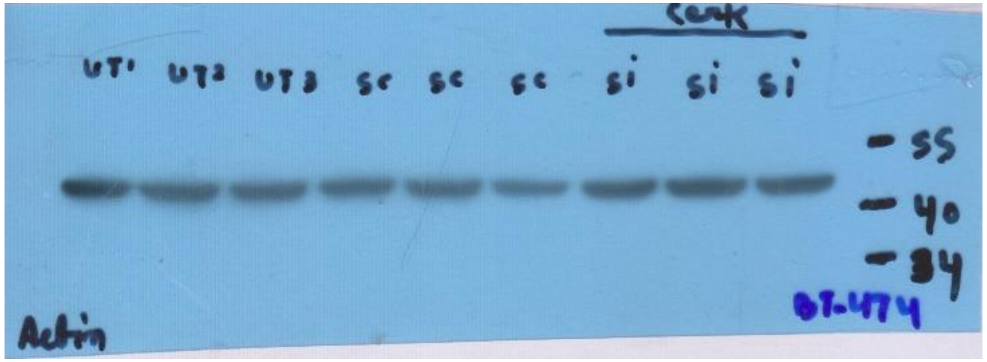

CerK siRNA in MDAMB-231

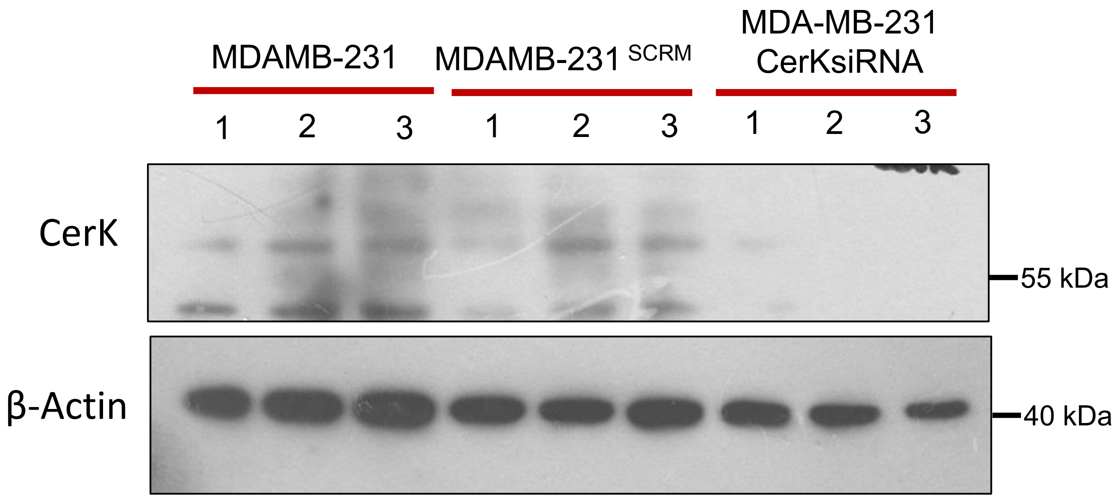

CerK siRNA in 231
Original Blot

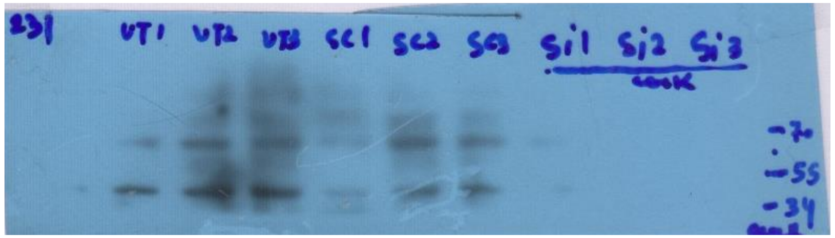

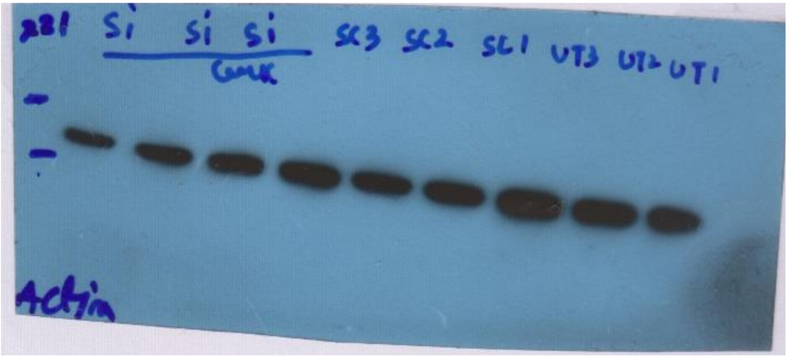

Supplement: Supplementary file 1 [file cancers-14-04496-s001.zip › cancers-1832882-supplementary/cancers-1832882-supplementary-Figure S4.pdf]
