# Supplementary material for: Ceramide Kinase (CERK) Emerges as a Common Therapeutic Target for Triple Positive and Triple Negative Breast Cancer Cells
Source: Cancers (Basel). 2022 Sep 16;14(18):4496. doi: 10.3390/cancers14184496 (PMC9497187; doi:10.3390/cancers14184496)
Supplement: Supplementary file 1 [file cancers-14-04496-s001.zip › cancers-1832882-supplementary/cancers-1832882-supplementary.pdf]

Supporting Information

Ceramide Kinase (CERK) Emerges as a Common Therapeutic Target for Triple Positive and Triple Negative Breast Cancer Cells.

Kajal Rajput,[1,a] Mohd. Nafees Ansari,[1,a] Somesh K. Jha,[2,a] Trishna Pani,[1] Nihal Medatwal,[2] Somdeb Chattopadhyay,[2] Avinash Bajaj,[2,*] and Ujjaini Dasgupta[1,*]

1. Amity Institute of Integrative Sciences and Health, Amity University Haryana, Panchgaon, Manesar, Gurgaon-122413, Haryana, India.
2. Regional Centre for Biotechnology, NCR Biotech Science Cluster, 3rd Milestone Faridabad-Gurgaon Expressway, Faridabad-121001, Haryana, India.

a: These authors contributed equally to the work.

Corresponding authors:

Email: bajaj@rcb.res.in

udasgupta@ggn.amity.edu

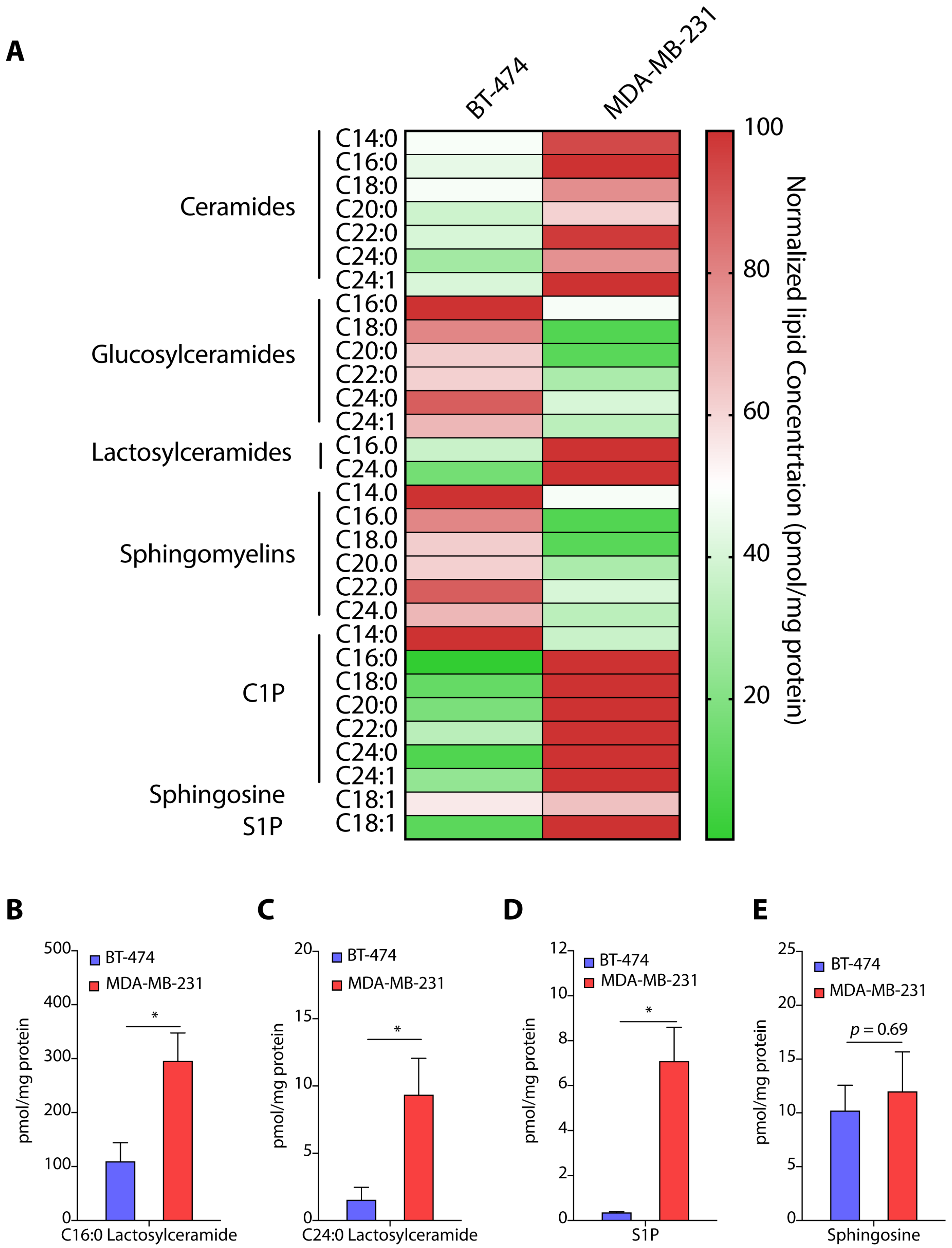

Figure S1. (A) Heatmap showing the levels of different sphingolipids in BT-474 and MDA-MB-231 cells. **(B-E)** Absolute quantification of C16:0 lactosylceramides (**A**), C24:0 lactosylceramides **(B)**, S1P **(C)**, and sphingosine **(D)** in BT-474 and MDA-MB-231 cells. Data is presented as mean ± SEM of six independent replicates, and was analysed using unpaired student's *t*-test. . *p*-value: $*p < 0.05$.

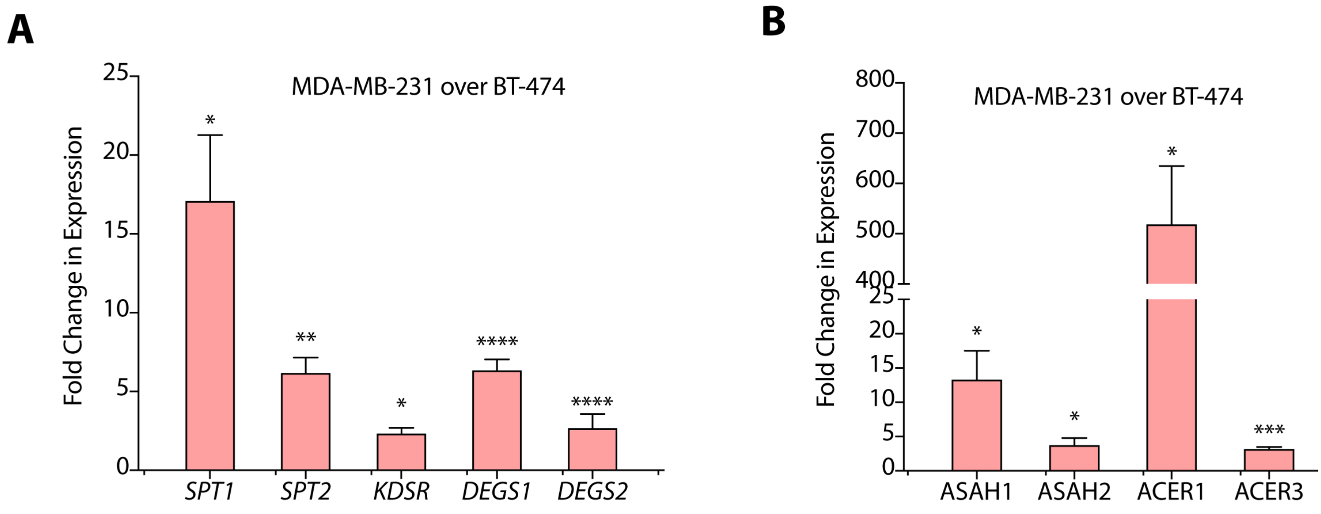

Figure S2. Gene expression of different sphingolipid-metabolizing genes in MDA-MB-231 cells as compared to BT-474 cells. Data is presented as mean ± SEM of at least three independent replicates, and was analysed using unpaired student's *t*-test. *p*-value: *p < 0. 05, **p < 0.01, ****p < 0.0001.

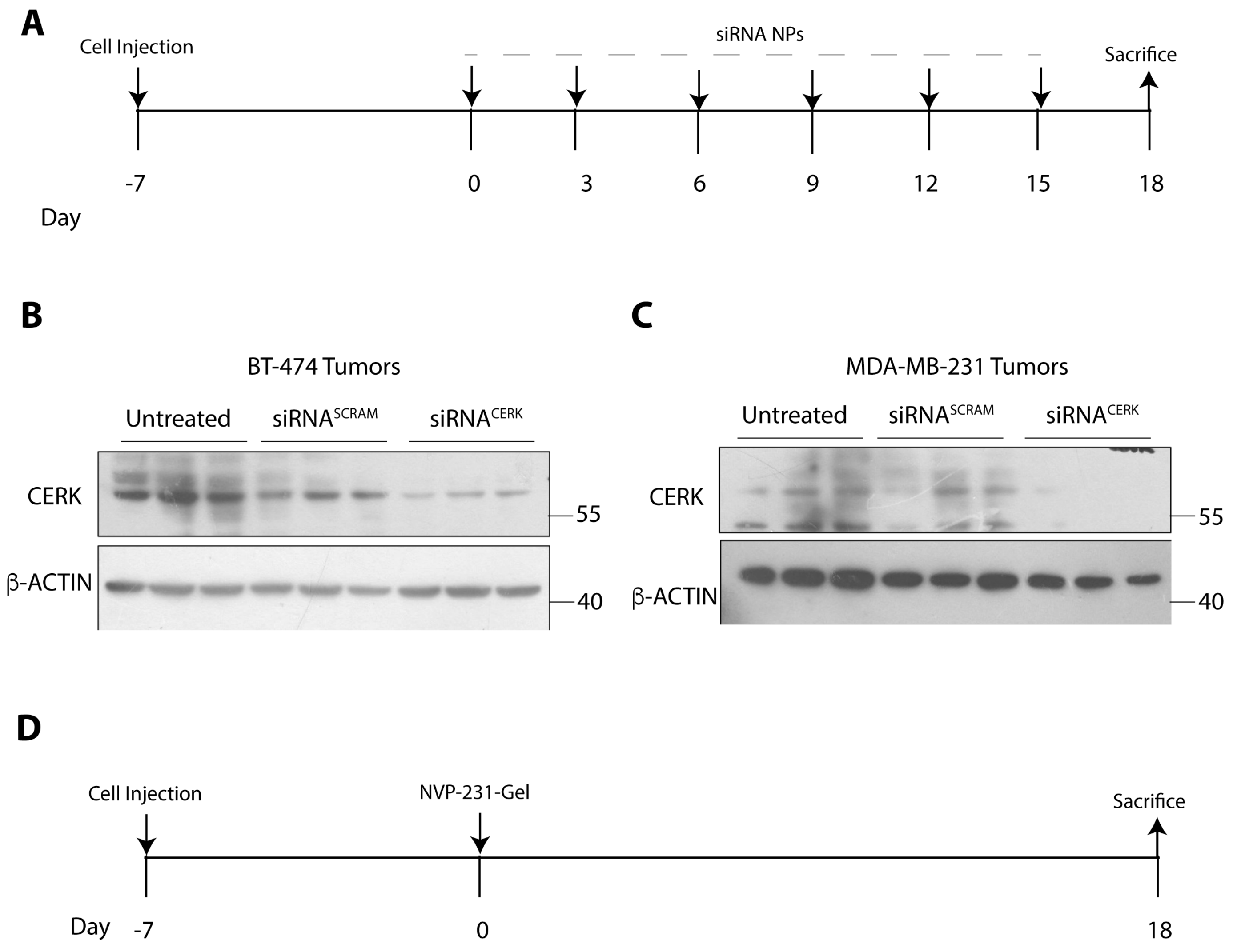

Figure S3. **(A)** Schematic showing the experimental design to decipher the effect of siRNA-mediated knockdown of CERK on tumor progression. **(B, C)** Validation of knockdown of CERK siRNA in BT-474 **(B)** and MDA-MB-231 **(C)** tumor tissues by immunoblotting. **(D)** Schematic showing the experimental design to decipher the effect of NVP-231-Gel-mediated inhibition of CERK on tumor progression.

Supplementary Table S1. List of primers (human) used for validation of endogenous gene expression by Real-Time PCR.

Gene Name	Forward primer (5' to 3')	Reverse primer (5' to 3')
Sptlc1	GTTCATTTGGATTTGGAAGACCG	CAGGCAGCTCTATCTACAAAAAC
Sptlc2	GAGGCTCACAGCATTGGCG	GTCTATCAGCTCCTTCTTGCC
Kdsr	GCAGATGGAGGTGAAGCCAT	CGCTCCTTCATGGTGGTGAT
Degs1	GAAAGGAACGAGATTCTGGAG	CTTCCTCACCAGATGAAAAGAA
Degs2	GGAGATACTGGCCAAGTACC	CAGCGTCAGCGAGTGGTTCA
CerS1	GTCATGTGGCGCCTGTTTC	CCAGGAGGAGGAGGAGGT
CerS2	GCGCAA AATGAGGTAGGC C	GCGCAAAATGAGGTAGGCC
CerS3	CATCCTGGATGCTCTTCATG	CGAGGTGATACATAGGCAAG
CerS4	CTTCGTGGCGGTCATCCTG	TGTAACAGCAGCACCAGAGAG
CerS5	AGAACGTGAGCTGGGCTGAT	GGCAATAAATCGCTCGAAGAG
CerS6	GCTTTGTTGCTGACGTGGACC	CCACTATGCTGGGTACGAGC
Asah1	TTCAAACCAGGACTGTTCAGTC	GTTGCTCAGTTATGACCCAGG
Asah2	GGTATCTTGAAGAGCATTGAC	CATTATCTCTTCTGTACAGGG
Acer1	GGAGTACAGGAAGACCAGCA	GATGAAGACCAGGCGGATG
Acer3	GCCGAGTTCTGGAATACAGT	CTGTGAGTGCTAAATAAGAAGC
Sphk1	CTGATGCACGAGGTGGTGAAC	CAGGAGGTCTTCATTGGTGAC
Sphk2	CTCATCCAGACAGAACGACAG	CACAGGCATCTTCACAGCTTC
CerK	GAGAAGCTGACGTCCAGAC	GTCTCCTTGGCCTGATTAGC
Sgms1	GTTCTCACCACAGTGATGATC	CAGTTGGGCTTCCCAGTCTC
Sgms2	GTGTGCTCCAAAGCTCAATGG	GTCAGTGTCAGCGTAACCGT
Smpd1	CTCACCCTCTTTCTCCCCA	GTTCCTTCTTCAGCCCGAG
Smpd2	CTACCCACGGACCAGCAGA	CCCAGGCCAATCACATAGC
Smpd3	GCTTCAAGTGTCTCAACAGC	CCACCTGCACCTTGAGAAAC
Smpd4	GGCTGGGCTTTAGCTCCAT	CTTCGCTGAGCCGGAATATC
Ugcg	GAATGGCCGTCTTCGGGTTC	CACAAGAGAAGACACCTGGGAG
Gba1	GATACCAAGCTCAAGATACCC	GGTCTGGTGGTAGATGTCTC
Glb1	GAACGCCATCCAGACGTATG	GCCAAGCAGGTAATCCTCC
B4galt6	CCGGAACTATTACGGATGTGA	GTGTGCCAGTCTGTTCAATGA
β-actin	ATTGGCAATGAGCGGTTCC	GGTAGAGTTTCGTGGATGCCACA
